# Genome-Wide Analysis of *Fusarium oxysporum* f. sp. *cubense* Peroxidases Reveals Oxidative-Stress Responses and Infection-Associated Expression

**DOI:** 10.3390/jof12090653

**Published:** 2026-09-01

**Authors:** Zhaojian Ding, Han Ouyang, Yu Chen, Xinyi Lian, Huiqun Yang, Yangjiao Zhou, Huijiao Lin, Qiyan Fu

**Affiliations:** 1 Department of Biological Sciences, Qiongtai Normal University, Haikou 571127, China; 2 Tropical Biodiversity and Bioresource Utilization Laboratory, Qiongtai Normal University, Haikou 571127, China; 3 Tropical Agricultural College, Hainan College of Vocation and Technique, Haikou 570216, China

**Keywords:** *Fusarium oxysporum* f. sp. *cubense*, heme-dependent peroxidase, oxidative-stress response, infection-associated expression

## Abstract

Banana Fusarium wilt, caused by the soil-borne fungus *Fusarium oxysporum* f. sp. *cubense* (Foc), is a devastating disease and a major threat to global banana production. Here, we identified 24 genes predicted to encode heme-dependent peroxidase- or catalase–peroxidase-related proteins in Foc race 4 (Foc4), whereas nine genes encoding thiol-dependent peroxide-reducing proteins were catalogued separately. Phylogenetic, synteny, and Ka/Ks analyses indicated a conserved peroxidase repertoire under strong purifying selection, without substantial lineage-specific expansion. Motif, domain, gene-structure, and promoter analyses revealed subgroup-specific features and abundant putative stress- and hormone-responsive cis-regulatory motifs. Expression profiling during banana infection and H_2_O_2_ treatment showed distinct temporal and oxidative-stress responses. Gene Ontology (GO) and Kyoto Encyclopedia of Genes and Genomes (KEGG) analyses linked subsets of these genes to antioxidant activity, peroxide metabolism, peroxisomal functions, and stress signaling. A *F. oxysporum* f. sp. *lycopersici* ortholog-based interaction network combined with co-expression analysis prioritized five peroxidases potentially associated with pathogenicity-related expression programs. *FoCP*, selected separately based on its rapid H_2_O_2_ response and catalase–peroxidase annotation, enhanced oxidative-stress tolerance when heterologously expressed in yeast. Overall, Foc4 appears to adapt to oxidative stress through condition-specific regulation of a conserved peroxidase repertoire rather than gene-family expansion. The network-prioritized peroxidases provide candidates for further functional and pathogenicity studies.

## 1. Introduction

Banana (*Musa* spp.) is one of the most important tropical and subtropical crops worldwide, and its yield and quality directly influence food security and farmers’ incomes. Since the late 20th century, banana Fusarium wilt has posed a severe threat to the global banana industry [1]. This disease is caused by the soil-borne fungal pathogen *Fusarium oxysporum* f. sp. *cubense* (Foc) and can rapidly colonize banana plants, inducing whole-plant wilting and mortality. Among Foc, race 4 (Foc4) infects nearly all banana cultivars and is highly virulent on Cavendish, and can persist in soil for years in the absence of host plants, posing a sustained and serious threat to global banana production [2,3,4]. Given the high tolerance of Foc4 to chemical control and the limited availability of resistant cultivars, elucidating the pathogenic mechanisms of Foc4 is crucial for informing the development of new management strategies. *Fusarium* infection in bananas triggers increased reactive oxygen species (ROS) and changes in antioxidant enzyme activities, particularly in roots, stems, leaves, and fruits, suggesting activation of strong plant defense responses, potentially including HR-associated processes [5]. However, despite the global impact, the molecular determinants of Foc4 virulence, particularly the networks governing ROS detoxification during host colonization, remain poorly understood.

During plant–pathogen interactions, ROS play dual roles as defense signaling molecules and as a major barrier that pathogens must overcome [6,7]. Upon invasion, ROS such as hydrogen peroxide (H_2_O_2_) rapidly accumulate as an early defense signal, generating an oxidative burst that restricts pathogen spread and proliferation and can trigger programmed cell death, thereby contributing to local resistance [8]. However, excessive or sustained ROS accumulation may also cause host tissue damage and influence disease progression, particularly during late infection stages [9]. Many pathogenic fungi have evolved diverse antioxidant systems to detoxify and scavenge ROS, thereby weakening plant defenses and facilitating successful infection [10,11].

Peroxidases constitute a functionally diverse core family of antioxidant enzymes in fungi, playing multifaceted roles in ROS detoxification, redox signaling, and host–pathogen interactions [8,12]. By catalyzing the reduction of H_2_O_2_ and other peroxides, they limit the reactivity of oxidants and contribute to intracellular redox homeostasis, thereby modulating oxidative stress responses and redox-dependent signaling processes that are closely associated with fungal virulence and survival under host-derived oxidative bursts [13]. From a plant–pathogen interaction perspective, fungal peroxide-reducing enzymes can be broadly categorized by catalytic cofactor/mechanism into heme-dependent peroxidases and thiol-dependent peroxidases. Heme-dependent peroxidases include DyP-type peroxidases (often annotated as EfeB/YfeX-like proteins), which participate in lignin degradation and have been implicated in host cell wall modification during plant–fungal interactions [14,15,16]; bifunctional catalase–peroxidases (KatG), which efficiently scavenge high concentrations of H_2_O_2_ and protect fungal cells during the early stages of infection [17,18]; and ascorbate peroxidases (APX), which help maintain ROS homeostasis in specific cellular compartments and, in some pathosystems, can act as secreted virulence factors targeting host organelles [19]. In parallel, thiol-dependent peroxidases, represented by peroxiredoxins (PRXs), not only reduce peroxides but also function as redox sensors and regulators of signaling pathways that tune fungal development and pathogenicity under fluctuating host-derived ROS pressure [13,20,21]. This mechanistic partitioning underscores that plant pathogenic fungi must coordinate both extracellular/apoplastic ROS detoxification and intracellular redox signaling to adapt to the dynamic oxidative environment during host colonization.

The biological relevance of these peroxidase subfamilies has been substantiated by functional studies in diverse plant-pathogenic fungi. For example, the DyP-type peroxidase BcPRD7 in *Botrytis cinerea* contributes to oxidative stress tolerance and maintenance of cell-wall integrity [14]. In *F. graminearum*, the bifunctional enzyme encoded by *KatG2* is strongly induced under H_2_O_2_ stress and localizes to the cell wall of infection hyphae; through spatiotemporally regulated scavenging of host-derived ROS at the infection site, it maintains a low-oxidation microenvironment that promotes penetration and colonization [8]. *MoApx1*, an *APX* family member, not only neutralizes H_2_O_2_ but also targets the rice photosystem I subunit OsPsaD and binds starch, thereby suppressing host immunity and perturbing energy supply [19]. Furthermore, the core PRX enzyme MoPRX1 in *Magnaporthe oryzae* scavenges host ROS during the early infection stage; its deletion leads to substantial H_2_O_2_ accumulation in host cells, highlighting the role of the peroxidase network in alleviating oxidative stress [12]. Consistent with these gene-specific observations, genome-wide and functional phenotypic analyses similarly show that deletion mutants of peroxidase-related genes in *F. graminearum* and *B. cinerea* exhibit heightened sensitivity to H_2_O_2_ and reduced virulence [14,22]. Collectively, peroxidases play critical roles in fungal pathogenesis and represent potential targets for controlling a broad spectrum of plant diseases.

In the present study, we performed a genome-wide identification and systematic characterization of the peroxidase gene family in Foc4. Phylogenetic, structural, evolutionary, functional annotation, and promoter analyses were conducted to characterize this gene family. Transcriptomic data from banana infection stages and H_2_O_2_ treatment, together with qRT-PCR validation, were used to investigate the expression patterns of selected genes. In addition, network and co-expression analyses were performed to identify peroxidases potentially associated with infection-related transcriptional programs, and heterologous expression in *Saccharomyces cerevisiae* was used to evaluate the contribution of a selected catalase–peroxidase candidate to oxidative-stress tolerance.

## 2. Materials and Methods

### 2.1. Identification and Characterization of Peroxidase Gene Family

Genome sequences and GFF3 annotation files of the Foc4 strain II5, together with RNA-seq data obtained from Foc4-infected banana roots at different time points, were obtained from the NCBI database (BioProjects PRJNA859936 and PRJNA1113144, accessed on 6 October 2025) [23]. These datasets were used for genome-wide identification of peroxidase genes and infection-related expression profiling. In addition to these publicly available datasets, we generated an independent H_2_O_2_-stress RNA-seq dataset from a Chinese Foc4 isolate in the present study. This experimentally generated dataset was used to evaluate the transcriptional responses of Foc4 peroxidase genes to oxidative stress. Clean reads were mapped to the Foc4 strain II5 reference genome; consequently, all genes in the H_2_O_2_-stress analysis are reported using II5 annotation identifiers. Conserved domain hidden Markov model (HMM) profiles associated with peroxidases were retrieved from the Pfam database and used to identify candidate peroxidase proteins from the Foc4 proteome using TBtools v2.388 [24]. The presence of conserved peroxidase-related domains was further verified using the NCBI Conserved Domain Database (CDD) through the CD-Search tool (accessed on 10 October 2025), together with the Peroxiscan platform (accessed on 10 October 2025) [25], and proteins lacking characteristic peroxidase-related domains were excluded from subsequent analyses. For clarity, standardized gene identifiers were used throughout this study, which correspond to the original gene IDs in the genome annotation (GFF3) file.

Basic physicochemical properties of the identified peroxidase proteins, including protein length, molecular weight, and theoretical isoelectric point, were predicted using the ExPASy ProtParam tool (https://web.expasy.org/protparam/, accessed on 2 December 2025). Subcellular localization was predicted using WoLF PSORT (https://wolfpsort.hgc.jp/, accessed on 2 December 2025). Conserved motifs were identified using the MEME program with default parameters (https://meme-suite.org/meme/, accessed on 19 December 2025), and exon–intron structures were extracted from the GFF annotation file and visualized using TBtools.

### 2.2. Phylogenetics

To investigate the evolutionary relationships among peroxidase proteins, amino acid sequences of all identified peroxidases from *F. oxysporum* f. sp. *lycopersici* (Fol), *F. graminearum*, and *M. oryzae* were collected. These sequences, together with the peroxidases identified in Foc4, were subjected to phylogenetic analysis. Multiple sequence alignment was carried out using ClustalW (version 2.1), followed by trimming with trimAl (-automated1) to remove poorly aligned and gap-rich regions, retaining 453 of 1286 aligned sites. Model selection and maximum-likelihood inference were performed using IQ-TREE (v2.3.6). The WAG+G model (with a discrete gamma distribution of four rate categories for among-site rate heterogeneity) was identified as the best-fit model according to the Bayesian information criterion (BIC) implemented in ModelFinder. Branch support was assessed using 1000 ultrafast bootstrap (UFBoot) replicates, and only bootstrap values ≥ 50% were displayed. The final phylogenetic tree was visualized and annotated using iTOL.

To evaluate the selective constraints acting on peroxidase genes, pairwise nonsynonymous (Ka) and synonymous (Ks) substitution rates were estimated. Coding sequences of orthologous peroxidase genes were aligned according to their corresponding protein sequence alignments. The Ka, Ks, and Ka/Ks values were calculated using the Ka/Ks Calculator implemented in TBtools. Genes exhibiting Ka/Ks ratios greater than 1 were inferred to be under potential positive selection, whereas ratios below 1 were indicative of purifying selection.

### 2.3. Chromosomal Localization and Synteny Analysis of Peroxidase Genes

The 11 Foc4 chromosome sequences were re-labeled as Chr1–Chr11 according to their GenBank deposition order. Gene coordinates were extracted from the GFF3 file and visualized using TBtools (using the Gene Location Visualize from GTF/GFF module). For genes with multiple transcript isoforms, a single representative gene locus was used for subsequent analyses. To identify gene duplication patterns, synteny analyses of peroxidase genes were conducted by comparing Foc4 with Fol and *F. oxysporum* Fo47 (Fo47). These analyses were performed using the Dual Synteny Plot program, which integrates the MCScanX algorithm, within the TBtools software platform. For the MCScanX analysis [26], BLASTp (BLAST+ v2.17.0) alignments were conducted with an E-value cutoff of 1 × 10^−3^, and the top 10 matches were retained for collinearity analysis.

### 2.4. Analysis of Cis-Acting Elements in the Peroxidase Gene Family

The 2000 bp upstream promoter sequences corresponding to the annotated peroxidase genes of Foc4 were retrieved using the Gff3 Sequences Extractor module in TBtools. Putative cis-acting regulatory element-like motifs within these promoter regions were subsequently identified using the PlantCARE database (http://bioinformatics.psb.u-gent.be/webtools/plantcare/html/, accessed on 28 December 2025) [27].

### 2.5. Dynamic Expression Profiling of Foc4 Peroxidase Genes During Banana Host Infection

To analyze the dynamic expression of Foc4 peroxidase genes during infection of banana roots, fragments per kilobase of transcript per million mapped reads (FPKM) values derived from RNA-seq data for the peroxidase genes detected in the infection-stage transcriptome were obtained at three time points (18, 32, and 56 h). The FPKM values were first log2-transformed as log2 (FPKM + 1), and heatmaps with hierarchical clustering were generated in R (v4.4.2) using the pheatmap package. Row-wise Z-score scaling was performed within pheatmap by setting scale = “row”.

### 2.6. Expression Profile of Peroxidase Genes Under H_2_O_2_ Stress

To investigate the response of peroxidase genes to H_2_O_2_ stress, fresh Foc4 conidia from a Chinese Foc4 isolate (1 mL of 1 × 10^8^ conidia·mL^−1^) were inoculated into 100 mL potato dextrose broth (PDB) and cultured at 28 °C with shaking at 180 rpm for 18 h; hydrogen peroxide (H_2_O_2_) was then added to a final concentration of 1.5 mM, and samples were collected at 0, 0.5, 1, 2, 4, and 8 h after treatment for transcriptome sequencing. Samples collected at 0, 0.5, 2, and 8 h were used for quantitative real-time PCR (qRT-PCR) validation. The 1.5 mM concentration was selected as a sublethal dose on the basis of preliminary dose–response experiments, in which 3–4 mM H_2_O_2_ markedly inhibited Foc4 growth on plates at 28 °C over 3 days, and it falls within the range (1–5 mM) commonly used for sublethal H_2_O_2_ treatment and transcriptional profiling in plant-pathogenic fungi [8,12,22].

Fresh mycelia were harvested at each time point (with three biological replicates per time point), immediately frozen in liquid nitrogen, and stored at −80 °C until RNA extraction. RNA-seq libraries were prepared and sequenced on an Illumina NovaSeq platform (2 × 150 bp paired-end reads). Raw reads were processed with fastp to remove adapter sequences, poly-N reads, and low-quality reads, and Q20, Q30, and GC content were calculated. Clean reads were mapped to the Foc4 strain II5 reference genome using HISAT2 (v2.2.1), and gene-level raw counts were generated with featureCounts (v2.0.6); FPKM values were then calculated based on gene length and mapped read counts. Differential expression analysis was performed on raw read counts using DESeq2; genes with |log2 fold change| ≥ 1 and adjusted *p* value < 0.05 were considered differentially expressed. To visualize peroxidase gene responses to H_2_O_2_, FPKM values for peroxidase genes were transformed as log2 (FPKM + 1). Heatmaps with hierarchical clustering were generated in R using the pheatmap package with row wise Z score scaling (scale = “row”).

### 2.7. Reference Gene Stability Screening and qRT-PCR Validation

To select an appropriate internal reference gene for qRT-PCR normalization, total RNA was extracted from the H_2_O_2_-stressed mycelial samples collected at 0, 0.5, 2, and 8 h using the E.Z.N.A.^®^ Total RNA Kit I (Omega Bio-tek, Inc., Norcross, GA, USA). The RNA samples were reverse-transcribed using the PrimeScript™ IV RT reagent Kit (TaKaRa, Dalian, China), and the resulting cDNA served as the template for both reference gene stability screening and target gene expression analysis. qRT-PCR was performed on a Bio-Rad CFX96 system (Bio-Rad Laboratories, Inc., Hercules, CA, USA) using gene-specific primers listed in Table S7. Four candidate reference genes, namely *actin*, *GAPDH* (glyceraldehyde-3-phosphate dehydrogenase), *β-tubulin*, and *EF1α* (elongation factor 1-alpha), were evaluated. Amplification specificity was confirmed by melting curve analysis. The expression stability of the candidate reference genes was assessed using the RefFinder web-based tool (https://www.heartcure.com.au/reffinder/, accessed on 7 February 2026), which integrates the Genorm, NormFinder, BestKeeper, and comparative ΔCt methods. Relative expression levels were calculated using the 2^−^^ΔΔCt^ method. Statistical significance of relative expression was assessed by one-way ANOVA followed by Tukey’s HSD test (*p* < 0.05).

### 2.8. Heterologous Expression in Saccharomyces cerevisiae and Assessment of H_2_O_2_ Tolerance

For functional characterization, one member of the putative peroxidase gene family was selected for heterologous expression in *S. cerevisiae*. The full-length coding sequence of the selected gene was amplified from Foc4 cDNA using gene-specific primers (Table S7) and inserted into the yeast expression vector pYES2-NTB (Invitrogen, Carlsbad, CA, USA) downstream of the galactose-inducible GAL1 promoter. The resulting recombinant construct was confirmed by sequencing and introduced into *S. cerevisiae* strain INVSc1 (Invitrogen, USA) using the lithium acetate transformation method. Transformants were selected on synthetic dropout agar medium lacking uracil (SD/-Ura) at 30 °C for 2 d.

To assess H_2_O_2_ tolerance, two independent transformants carrying the recombinant construct and one transformant carrying the empty pYES2-NTB vector were cultured in SG/-Ura liquid medium supplemented with 2% (*w*/*v*) galactose at 30 °C with shaking at 180 rpm for 48 h. The cultures were adjusted to an OD_600_ of 0.1 and subjected to 10-fold serial dilution from 10^0^–10^−3^. Aliquots (5 μL) of each dilution were spotted onto SG/-Ura agar plates containing 2% (*w*/*v*) galactose and 0, 1, or 3 mM H_2_O_2_. The plates were incubated at 30 °C for 3 d and then photographed. The assay was repeated independently three times.

### 2.9. GO and KEGG Pathway Analysis of Foc4 Peroxidase Genes

GO annotation and KEGG pathway enrichment analyses were performed for peroxidase genes expressed during banana host infection using the OmicShare Tools cloud platform (https://www.omicshare.com/tools/, accessed on 23 February 2026). Peroxidase genes expressed during infection were defined as the target gene set, while all Foc4 genes with assigned GO annotations (*n* = 6334) were used as the background gene set for GO functional enrichment analysis. Statistical significance of enrichment was determined using hypergeometric tests, and the resulting *p* values were corrected for multiple testing using the Benjamini–Hochberg false discovery rate (FDR) method; terms with corrected *p* (q-value) < 0.05 were considered significantly enriched.

For KEGG pathway enrichment analysis, peroxidase genes with assigned KEGG Orthology annotations were used as the target gene set, while all Foc4 genes included in the KEGG annotation pipeline were used as the background gene set. Hypergeometric tests were applied to identify significantly enriched pathways relative to the background, and pathways with FDR-corrected *p* (q-value) < 0.05 were considered statistically significant. The enrichment results were visualized using various charts and pathway maps generated by the OmicShare platform, providing an intuitive overview of the functional categories and metabolic pathways associated with the target gene set.

### 2.10. Construction and Analysis of Protein–Protein Interaction (PPI) Networks for Hub Peroxidases

Due to the limited annotation of the Foc4 strain in the STRING database, *F. oxysporum* f. sp. *lycopersici* (Fol) was used as a reference species for PPI prediction. Homologous proteins of the Foc4 peroxidases were identified in Fol using BLASTp with an E-value threshold of 1 × 10^−5^, and the best hit retained for each Foc4 peroxidase; these were considered best-hit homologs (putative orthologs) rather than confirmed one-to-one orthologs. The corresponding Fol homologs were submitted to the STRING database (https://string-db.org, accessed on 22 March 2026) to construct the PPI network, with the minimum required interaction score set to medium confidence (score ≥ 0.4). The resulting interaction data were imported into Cytoscape v3.10.4 for visualization and network analysis. Node topological parameters were calculated, and hub nodes were ranked using the Degree algorithm implemented in the cytoHubba plugin.

### 2.11. Co-Expression Analysis of Hub Peroxidase Genes and Pathogenicity-Related Genes

To identify potential associations between hub peroxidases and pathogenicity-related genes, co-expression analysis was performed using banana root infection transcriptome data. Hub peroxidase genes identified from the PPI network were further filtered based on their detectable expression during banana root infection.

Homologs of 23 pathogenicity-related genes from *F. oxysporum* and *F. graminearum* were identified based on records in the Pathogen–Host Interactions (PHI) database. These genes were differentially upregulated at 32 or 56 h post-inoculation (hpi) relative to 18 hpi, using thresholds of |log_2_ fold change| > 1 and FDR < 0.05. Mean FPKM values of the expressed peroxidase genes and the 23 PHI gene homologs were extracted from transcriptomes at 18, 32, and 56 hpi. Pearson correlation coefficients were calculated between the mean FPKM values at the three time points to evaluate co-expression relationships, and the resulting co-expression network was visualized using Cytoscape v3.10.4.

## 3. Results

### 3.1. Identification and Basic Characteristics of the Foc4 Peroxidase Gene Family

Based on the predicted protein sequences of the Foc4 genome, Pfam HMM profiles corresponding to the catalytic cores of heme-dependent peroxidases, including PF01328, PF04261, PF01266, PF00199, and PF00141, were used to perform an initial search for candidate peroxidase proteins with the Advanced HMMER tool in TBtools. The resulting candidates were further validated using the NCBI CDD and Peroxiscan. Finally, 24 proteins containing conserved domains characteristic of heme-dependent peroxidases or catalase–peroxidase-related proteins were retained. These 24 proteins were collectively defined as the Foc4 peroxidase gene family and were used in the subsequent gene family analyses. According to their conserved domains, these proteins comprised Peroxidase_2/Peroxidase_2 superfamily members, a DyP-type peroxidase annotated as EfeB (YfeX-like), ascorbate_peroxidase, plant_peroxidase_like superfamily proteins, and catalase-related proteins, including catalase_fungal/catalase_like superfamily/Catalase and KatG. The basic characteristics of these genes are summarized in Table 1, including protein length, molecular weight, theoretical pI, predicted subcellular localization, and conserved domain annotations. Notably, these peroxidase proteins exhibited substantial variation in protein length and predicted subcellular localization, reflecting the structural and functional diversity of the Foc4 peroxidase gene family.

In addition, we identified 9 non-heme, thiol-dependent peroxide-reducing proteins in the Foc4 genome, including eight peroxiredoxin/peroxiredoxin-like proteins (domains annotated as PRX_Typ2cys, PRX_1cys, PRX_BCP, PRX5_like, and PRX_like2) and one BtuE-like peroxidase (Table S1). Because these proteins are thiol-dependent reductases and do not harbor the canonical catalytic domains typical of heme-dependent peroxidases, they were not included among the 24 members of the Foc4 peroxidase gene family and were excluded from all subsequent gene family analyses unless otherwise specified. They are therefore reported separately in Table S1.

### 3.2. Phylogenetic Analysis of Peroxidase Proteins

**Figure 1:**
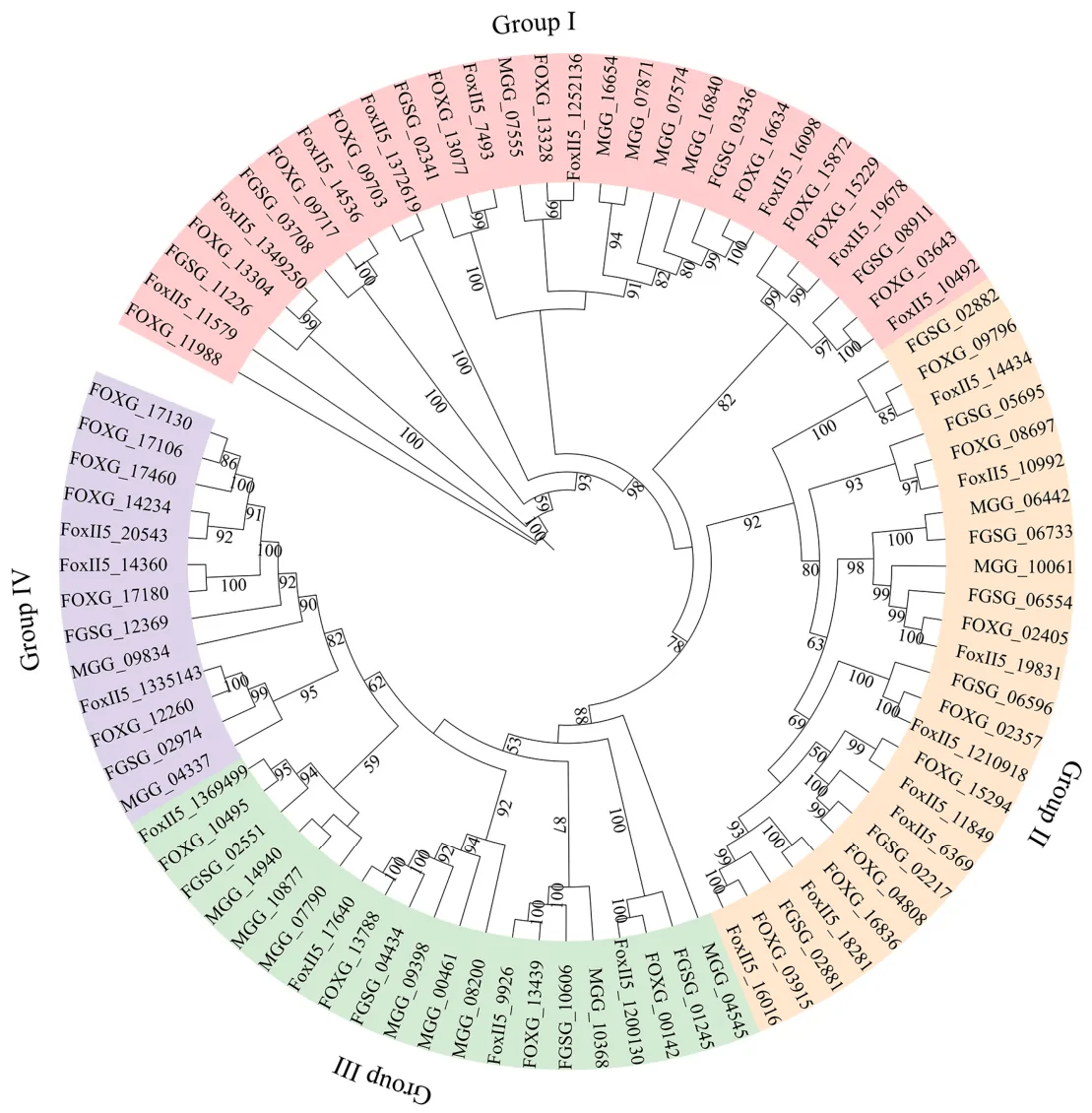
Maximum-likelihood phylogenetic analysis of peroxidase proteins from four plant-pathogenic fungi. Proteins are designated by species-specific prefixes: FoxII5_ for *Fusarium oxysporum* f. sp. *cubense* race 4, FOXG_ for *F. oxysporum* f. sp. *lycopersici*, FGSG_ for *F. graminearum*, and MGG_ for *Magnaporthe oryzae*. The identified peroxidases were classified into four phylogenetic groups (Groups I–IV).

Using full-length amino acid sequences, 24, 28, 18, and 17 peroxidase proteins from Foc4, Fol, *F. graminearum*, and *M. oryzae*, respectively, were assigned into four distinct groups (Figure 1, Table S2). Group I represents the core lineage of classical heme peroxidases. Proteins in this group primarily possess domains from the Peroxidase_2 or Peroxidase_2 superfamily, forming a robust monophyletic clade. Group II comprises proteins associated with catalases and atypical peroxidase-related proteins, including members of the catalase_fungal and catalase_like families, as well as katE/catalase-related proteins. In contrast, Group III includes plant_peroxidase_like superfamily proteins and ascorbate peroxidases (APXs). Group IV encompasses KatG-type catalase–peroxidases.

Phylogenetic analysis revealed nested clade patterns among peroxidase proteins across Foc4, Fol, *F. graminearum*, and *M. oryzae* (Figure 1). We identified 24 putative Foc4–Fol orthologous peroxidase protein pairs. For 23 of these pairs, the Fol ortholog clustered with the corresponding Foc4 protein. FoxII5_6369 clustered with the Fol protein FOXG_04808 and was also closely associated with the *F. graminearum* protein FGSG_02217. Ka/Ks analyses were conducted for the 24 Foc4–Fol orthologous pairs, whereas the FoxII5_6369–FGSG_02217 comparison was analyzed and reported separately (Table S3). For the 24 Foc4–Fol pairs, all Ka/Ks values were below 0.3 (mean = 0.081, median = 0.082, range = 0–0.295), and 22 pairs (91.7%) had Ka/Ks values below 0.2, indicating that most of these orthologous gene pairs have evolved under strong purifying selection. Across the four fungi examined, peroxidase gene counts ranged from 17 to 28 (Foc4: 24, Fol: 28, *F. graminearum*: 18, and *M. oryzae*: 17), with Fol differing from Foc4 by four genes (14.3% when Fol was used as the reference). These results indicate modest variation in gene copy number and the presence of a broadly conserved set of peroxidases across these plant-pathogenic fungi.

### 3.3. Structural Analysis of Peroxidase Gene Family

**Figure 2:**
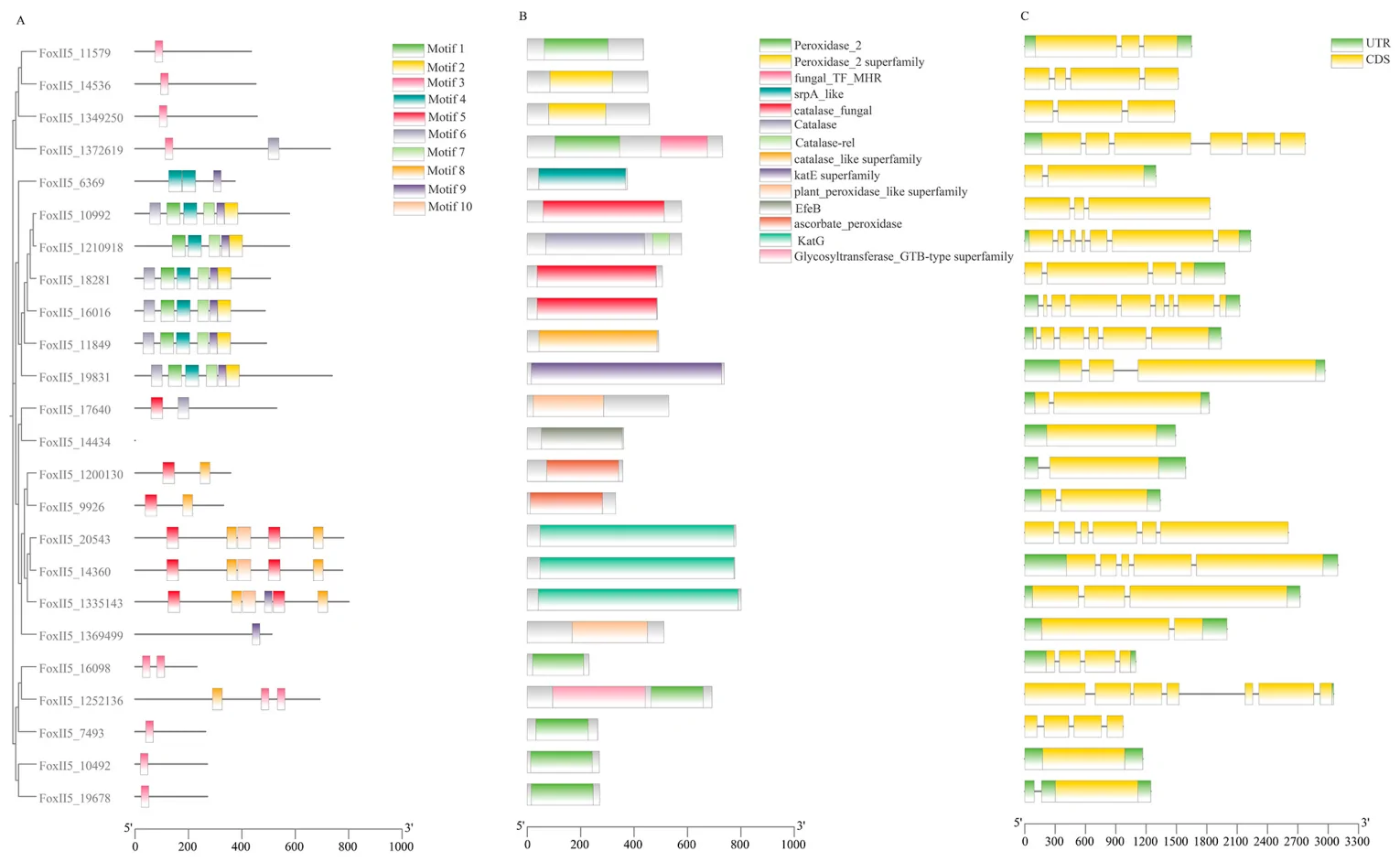
Comparative analysis of conserved motifs, domain architectures, and gene structures of the Foc4 peroxidase gene family. (**A**) Phylogenetic relationships of peroxidase proteins, accompanied by the distribution patterns of ten conserved motifs. (**B**) Conserved domains of peroxidase proteins predicted using the NCBI Conserved Domain Database (CDD), with different domains indicated by distinct colors. Scale bars represent protein length. (**C**) Exon–intron organization of peroxidase genes, where yellow boxes denote coding sequences (CDSs), black lines indicate introns, and green boxes represent untranslated regions (UTRs). The scale bar corresponds to genomic length.

Conserved motif analysis revealed distinct group-specific patterns (Figure 2A and Figure S1). In Group I, all peroxidases contained motif 3, whereas all Group II members contained motifs 4 and 9. Notably, six closely related Group II members, including *FoxII5_10992*, *FoxII5_1210918*, *FoxII5_18281*, *FoxII5_16016*, *FoxII5_11849*, and *FoxII5_19831*, shared five motifs (motifs 1, 2, 4, 7, and 9) with similar arrangements, indicating a highly conserved motif architecture. In Group III, with the exception of *FoxII5_1369499* and *FoxII5_14434*, the remaining six peroxidases shared several conserved motifs. Compared with the other groups, Group III exhibited greater variation in motif composition and arrangement, suggesting greater structural diversity among its members. No motif was detected in *FoxII5_14434*, possibly reflecting sequence divergence. The three Group IV peroxidases, *FoxII5_1335143*, *FoxII5_14360*, and *FoxII5_20543*, shared motifs 5, 8, and 10, indicating a conserved motif composition within this group.

In addition, genes belonging to the same subfamily generally exhibited comparable motif numbers and compositions. Conserved domain analysis further indicated that peroxidase members within each subfamily shared highly similar structural domain architectures (Figure 2B). By contrast, exon–intron organization varied substantially among individual peroxidase genes, and no clear subfamily-specific structural patterns were observed (Figure 2C).

### 3.4. Chromosomal Distribution and Synteny Analysis of Peroxidase Genes Across Fol, Fo47, and Foc4

**Figure 3:**
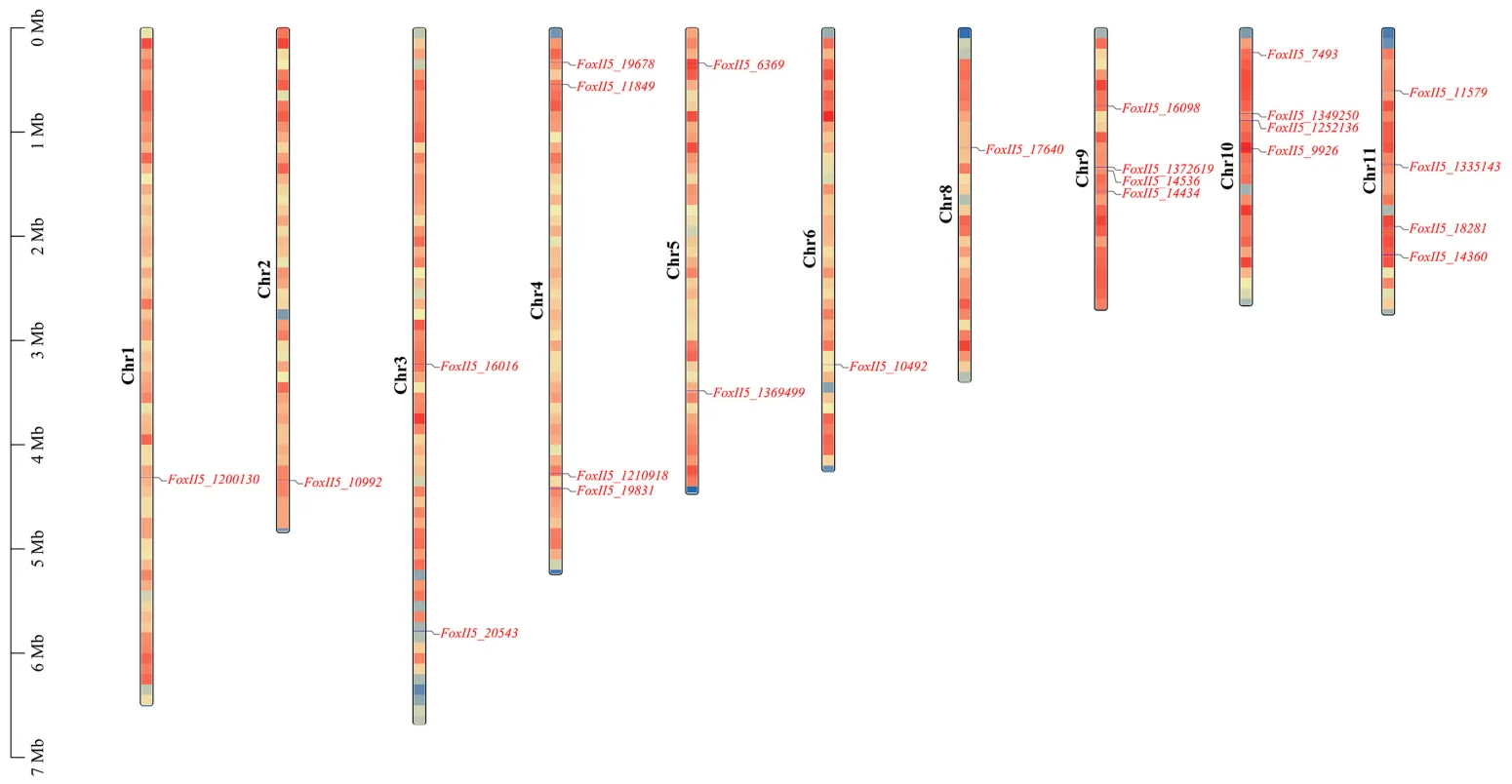
The 24 peroxidase genes are shown at their corresponding chromosomal positions. The vertical axis on the left represents chromosome length in megabases (Mb), while color gradients indicate overall gene density along each chromosome.

We mapped the chromosomal positions of 24 peroxidase genes to characterize their genomic distribution within Foc4 and assess patterns of gene clustering. These genes were distributed across 10 of the 11 chromosomes (Figure 3). Chromosomes 4, 9, 10, and 11 each harbored four genes; chromosomes 3 and 5 contained two genes; and chromosomes 1, 2, 6, and 8 contained one gene each. No peroxidase genes were identified on chromosome 7. All peroxidase genes were physically separated by distances ≥ 33 kb, indicating a dispersed genomic organization without tandem gene duplicates.

**Figure 4:**
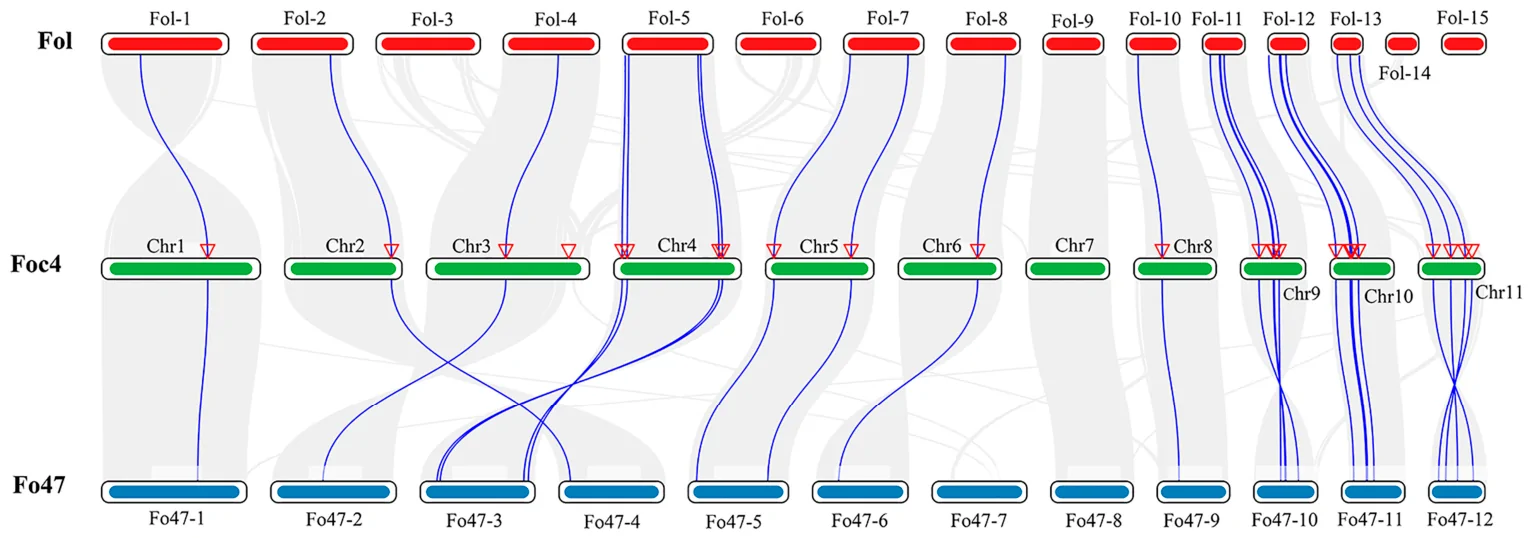
Comparative synteny analysis of peroxidase genes among *Fusarium oxysporum* f. sp. *lycopersici* (Fol), *F. oxysporum* Fo47 (Fo47), and *F. oxysporum* f. sp. *cubense* race 4 (Foc4). Gray lines represent conserved syntenic blocks, whereas blue lines link putative orthologous peroxidase gene pairs. Red triangles indicate the chromosomal locations of peroxidase orthologs. The connections represent computationally inferred syntenic relationships rather than experimentally validated functional associations.

Collinearity analysis revealed extensive conservation of the genomic positions and order of peroxidase genes between Fol and Foc4, as indicated by the clear, straight blue lines in Figure 4. By contrast, the non-pathogenic Fo47 exhibited frequent genome rearrangements and a more complex collinearity pattern relative to Fol and Foc4, evidenced by twisted and cross-linked connections. These results indicate greater conservation of the genomic regions harboring peroxidase genes between Fol and Foc4.

### 3.5. Sequence Analysis of Promoter Region of Peroxidase Gene Family

**Figure 5:**
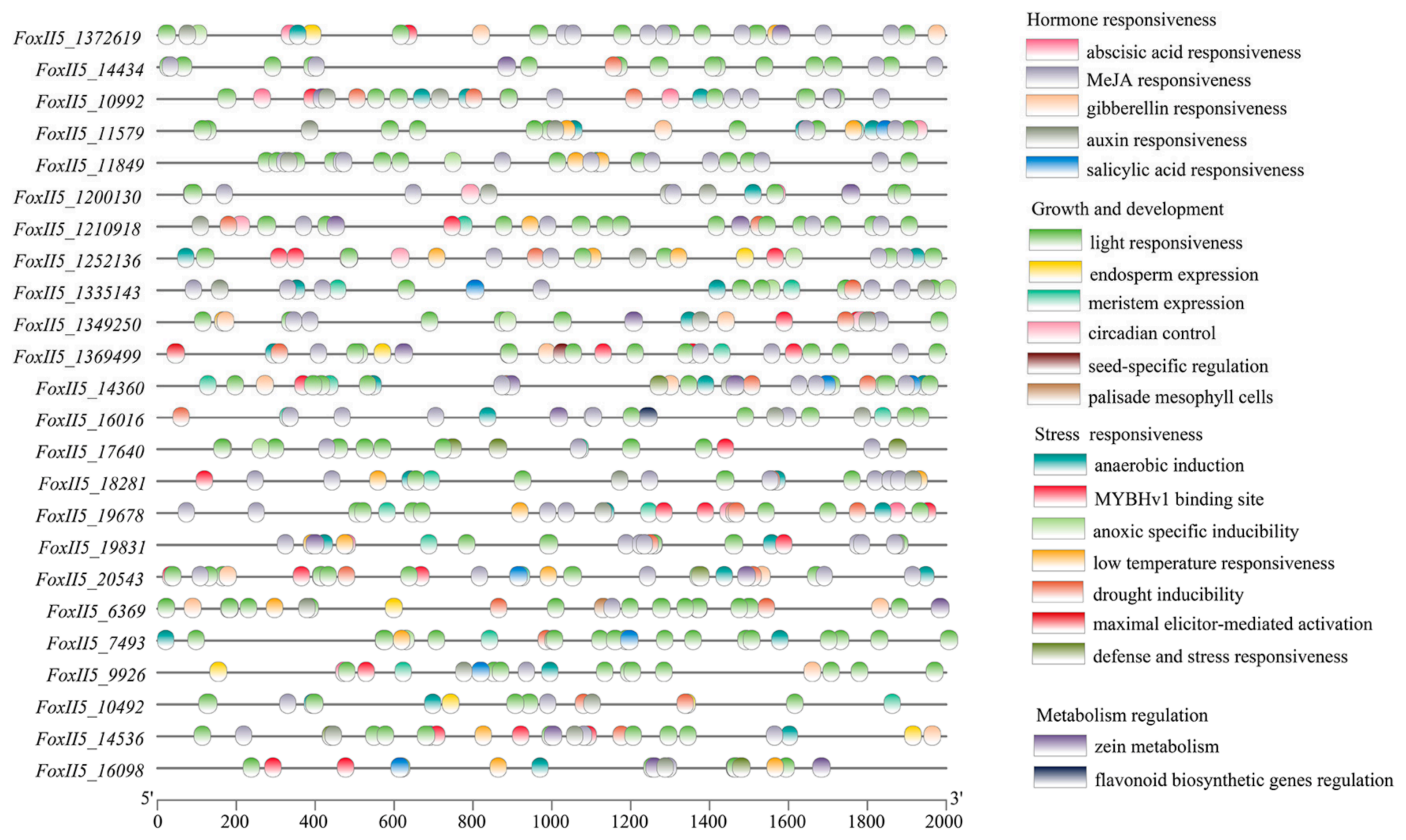
Analysis of cis-acting regulatory elements in the 2-kb upstream promoter regions of 24 peroxidase genes from *Fusarium oxysporum* f. sp. *cubense* race 4. Different cis-element types are indicated by color-coded boxes, as shown on the right. The scale bar represents promoter length in base pairs.

Putative cis-acting regulatory element-like motifs were analyzed in the 2.0 kb promoter regions upstream of the 24 identified peroxidase genes. A total of 695 putative regulatory motifs were detected and categorized into four functional groups: hormone responsiveness, growth and development, stress responsiveness, and metabolism regulation (Figure 5). Light-responsive elements were the most abundant individual element type (214), whereas MeJA-responsive (190) and ABA-responsive (58) elements predominated among the hormone-related elements (Table S4). Metabolism-related elements were comparatively less represented. Among stress-responsive elements, a notable subset comprising 43 hypoxia-related elements (including anaerobic induction and anoxic inducibility) was identified. These patterns suggest a potentially multilayered promoter architecture associated with environmental, developmental, hormonal, and metabolic signals. However, because PlantCARE is primarily a plant-oriented database, the identified motifs should be interpreted as putative sequence matches in Foc4, and their functional relevance requires experimental validation.

### 3.6. Dynamic Expression Analysis of Peroxidase Genes

**Figure 6:**
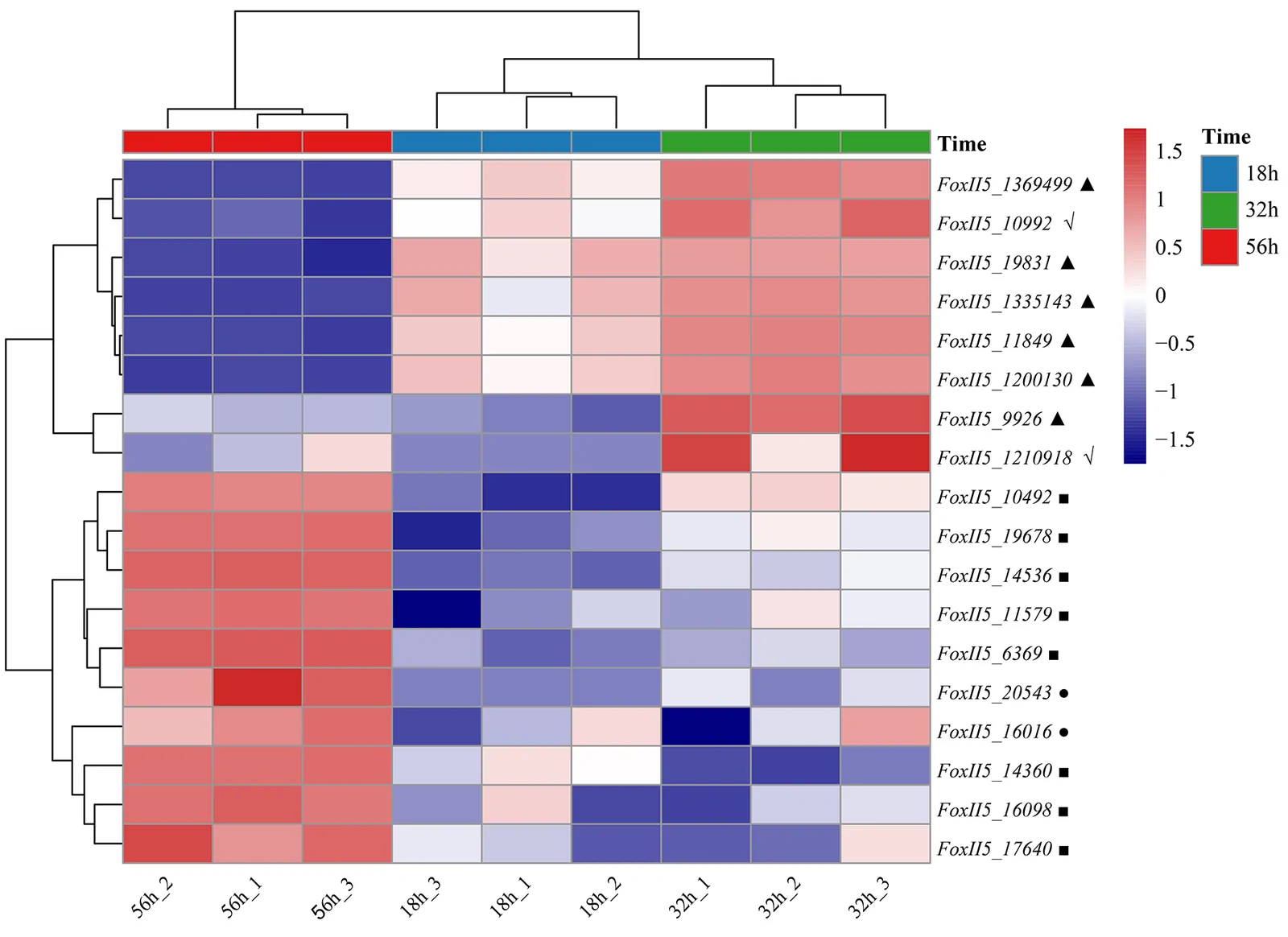
RNA-seq expression patterns of peroxidase genes in *Fusarium oxysporum* f. sp. *cubense* race 4 during banana infection. The heatmap shows row-scaled log2 (FPKM + 1) values for 18 peroxidase genes at 18, 32, and 56 h post-infection. The color gradient indicates standardized expression, with red indicating relatively high expression and blue indicating relatively low expression. Symbols denote expression categories: a filled triangle indicates early highly expressed genes; a filled square indicates late highly expressed genes; a check mark indicates weakly induced genes; and a filled circle indicates constitutively low-expressed genes. Hierarchical clustering was performed in R (v4.4.2) using the pheatmap package after row-wise Z-score normalization.

The temporal expression patterns of 18 of the 24 Foc4 peroxidase genes with available and detectable infection-stage expression data were analyzed at three post-infection time points: 18, 32, and 56 hpi. FPKM values were used to generate a heatmap illustrating their temporal expression patterns during banana infection (Figure 6, Table S5). Hierarchical clustering grouped the genes into three major expression clusters. Cluster I, the early high-expression group, contained six genes including *FoxII5_1335143*, *FoxII5_19831*, *FoxII5_11849*, and *FoxII5_1369499*. These genes showed relatively high expression at 18 h and reached their highest expression levels at 32 h, suggesting potential roles in early host–pathogen interactions. Cluster II, the late high-expression group, comprised eight genes including *FoxII5_17640* and *FoxII5_10492*. These genes showed relatively low expression at 18 and 32 h but were markedly more highly expressed at 56 h, indicating potential involvement in later infection stages. Cluster III, the low-expression group, included four genes with FPKM values below 10 at all three time points. This cluster was subdivided into weakly induced genes, such as *FoxII5_10992*, and constitutively low-expression genes, such as *FoxII5_16016*. Overall, the Foc4 peroxidase genes display distinct temporal expression patterns during banana infection, suggesting transcriptional specialization among family members in response to the changing host environment.

### 3.7. Reference Gene Stability Screening

The expression stability of four candidate reference genes—*actin*, *GAPDH*, *β-tubulin*, and *EF1α*—was evaluated under H_2_O_2_ stress conditions using RefFinder. The comprehensive analysis ranked *actin* as the most stably expressed reference gene (geometric mean = 1.19), followed by *EF1α* (1.86), *GAPDH* (2.83), and *β-tubulin* (3.22) (Table S8). Actin ranked first according to the comparative ΔCt, Genorm, and NormFinder methods and second according to BestKeeper, demonstrating consistent stability across the four evaluation algorithms. Accordingly, *actin* was used as the internal reference gene for all subsequent qRT-PCR analyses.

### 3.8. Peroxidase Gene Expression Dynamics Under H_2_O_2_ Stress

**Figure 7:**
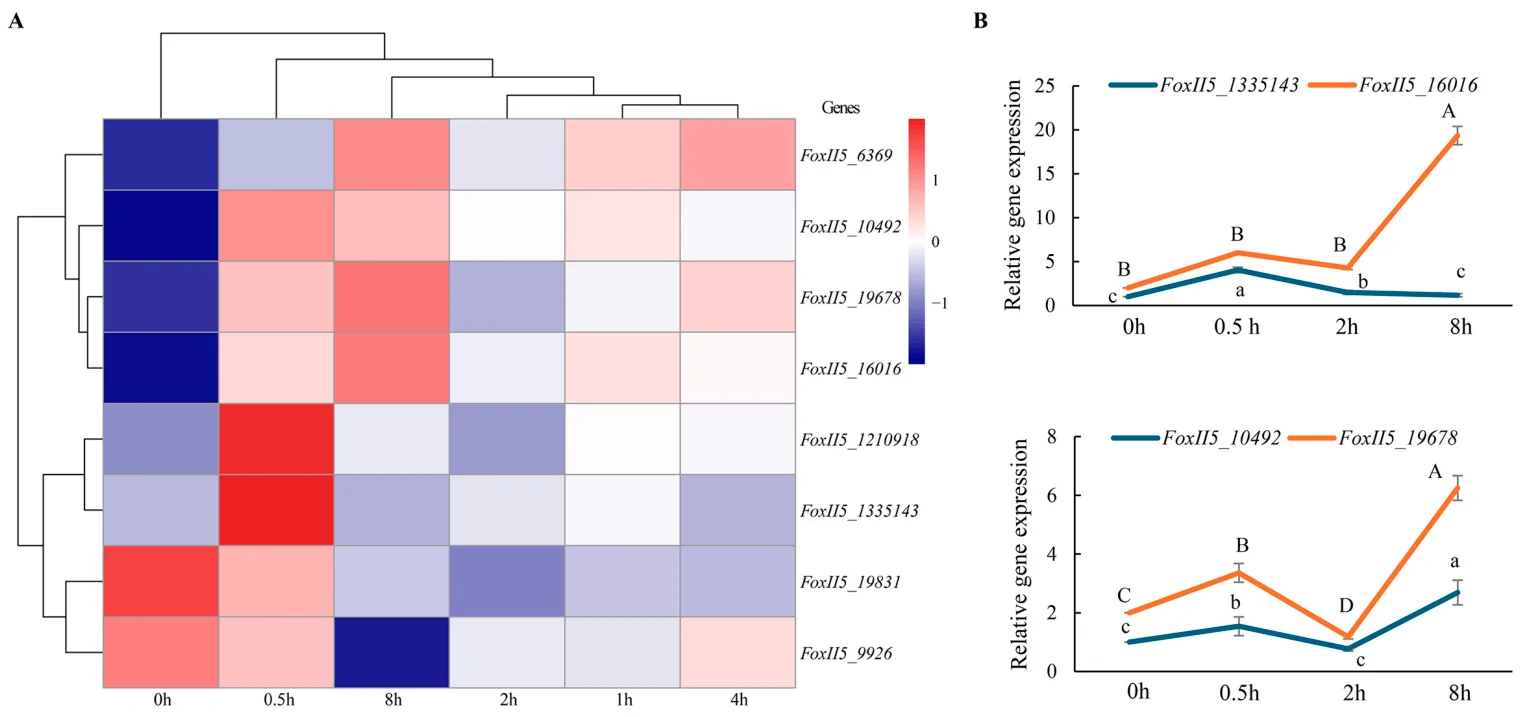
Expression profiles of *Fusarium oxysporum* f. sp. *cubense* race 4 peroxidase genes under H_2_O_2_ stress and qRT-PCR validation. (**A**) Heatmap of row scaled log2 (FPKM + 1) values for eight H_2_O_2_-responsive peroxidase genes across six time points from 0 to 8 h. Hierarchical clustering was performed with the R package pheatmap after row-wise Z-score normalization. (**B**) qRT-PCR validation of four representative peroxidase genes selected to cover the major H_2_O_2_-response patterns. Relative transcript levels were calculated using the 2^−ΔΔCt^ method, with *actin* as the internal reference gene. Error bars indicate the standard error of three replicates. Different letters indicate significant differences (one-way ANOVA followed by Tukey’s HSD test, *p* < 0.05); uppercase and lowercase letters denote different genes.

To investigate the transcriptional response of Foc4 to oxidative stress, we generated an RNA-seq dataset from a Chinese Foc4 isolate exposed to H_2_O_2_. Sequencing reads were mapped to the Foc4 strain II5 reference genome; therefore, genes are referred to by their corresponding II5 annotation IDs. Eight peroxidase genes displaying distinct expression patterns were examined in detail. *FoxII5_1335143* and *FoxII5_16016* showed rapid upregulation within 0.5 h, reaching 2.3- and 2.2-fold the corresponding levels at 0 h, respectively. *FoxII5_19678*, *FoxII5_10492*, and *FoxII5_6369* exhibited sustained upregulation from 2 h to 8 h, with *FoxII5_19678* showing the largest increase, from an FPKM value of 3.5 at 0 h to 15.7 at 8 h. *FoxII5_19831* and *FoxII5_9926* were down-regulated following H_2_O_2_ treatment, with expression decreasing steadily from 0.5 h onward. *FoxII5_1210918* remained at very low expression levels (FPKM < 0.27) across all time points, showing no notable response (Figure 7A, Table S6). Four representative peroxidase genes covering the major response patterns (*FoxII5_1335143*, *FoxII5_16016*, *FoxII5_10492*, and *FoxII5_19678*) were selected for qRT-PCR validation. The qRT-PCR expression trends were generally consistent with those obtained by RNA-seq (Figure 7B), supporting the reliability of the transcriptomic expression profiles (Figure 7B, Table S7).

When compared with their infection-stage profiles, *FoxII5_1335143* (early-induced) and *FoxII5_19678* (late-induced) displayed response kinetics that broadly paralleled their respective infection patterns; *FoxII5_19831* (early-induced but strongly down-regulated at 56 hpi) was repressed by H_2_O_2_; *FoxII5_16016* (low during infection) was rapidly induced by H_2_O_2_; *FoxII5_10492*, *FoxII5_6369*, and *FoxII5_1210918* showed low to very low H_2_O_2_-induced expression. These results suggest that H_2_O_2_ triggers distinct peroxidase expression programs, with several genes showing rapid or sustained induction, while others are repressed or remain unresponsive.

### 3.9. Heterologous Expression of FoCP in Yeast Confers H_2_O_2_ Tolerance

Among the eight peroxidase genes examined under H_2_O_2_ treatment, *FoxII5_1335143* (designated *FoCP*), which encodes a bifunctional catalase–peroxidase and exhibited a rapid early transcriptional response upon H_2_O_2_ exposure (Figure 7A), was selected for functional characterization. To assess whether *FoCP* could enhance tolerance to H_2_O_2_ stress, its full-length coding sequence was heterologously expressed in *S. cerevisiae* under the control of the GAL1 promoter.

**Figure 8:**
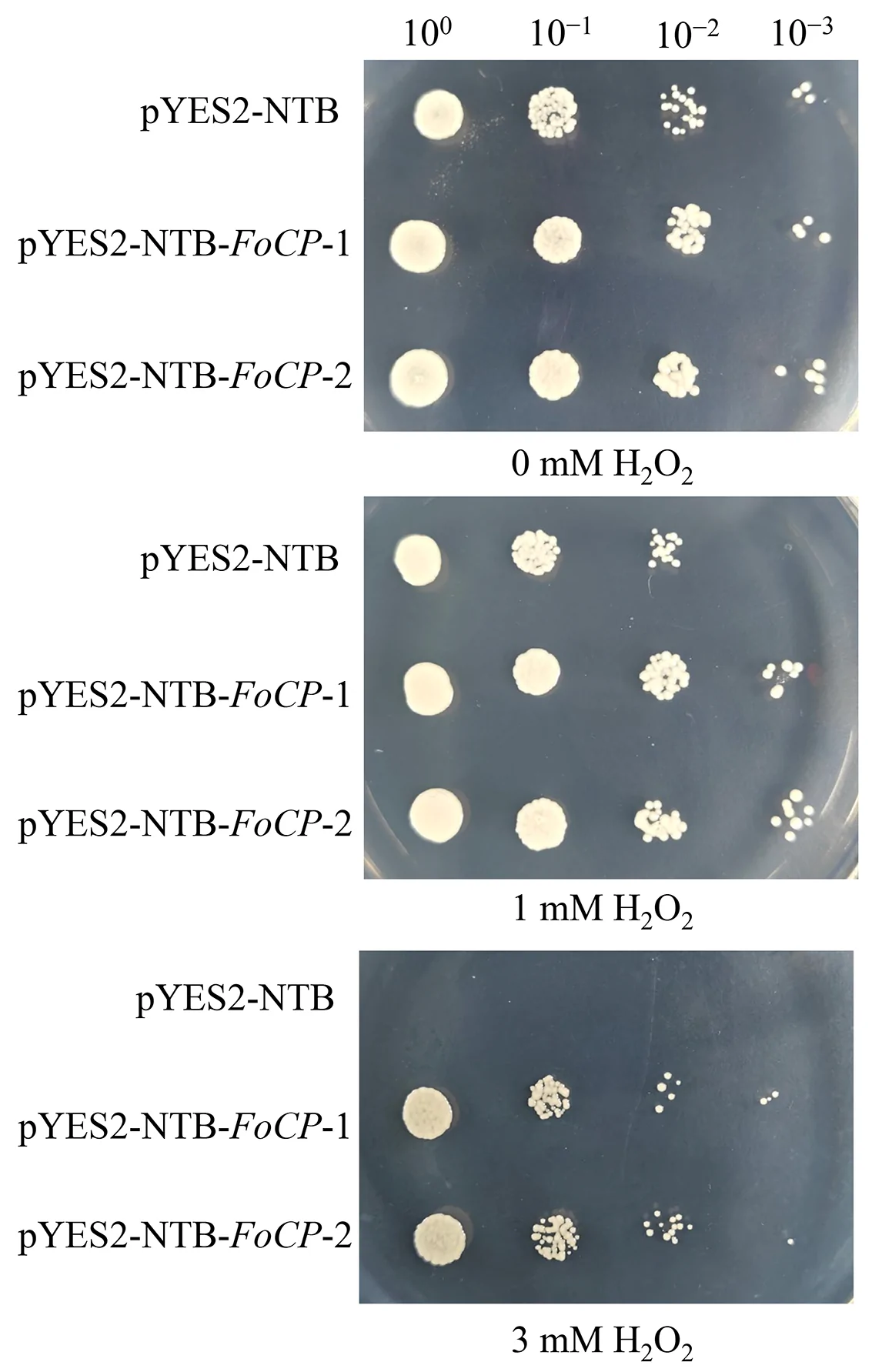
Heterologous expression of *FoCP* in *Saccharomyces cerevisiae* confers increased tolerance to H_2_O_2_ stress. Yeast cells carrying pYES2-NTB (empty vector) or either of two independently obtained *FoCP*-expressing transformants (pYES2-NTB-*FoCP*-1 and pYES2-NTB-*FoCP*-2) were cultured on SG/-Ura medium containing 2% galactose. Cultures were adjusted to OD_600_ = 0.1, and 10-fold serial dilutions (10^0^ to 10^−3^) were spotted onto SG/-Ura plates supplemented with 0, 1, or 3 mM H_2_O_2_. Plates were incubated at 30 °C for 3 days. The assay was performed as a semi-quantitative comparison.

On SG/-Ura control plates without H_2_O_2_, both the empty-vector control (pYES2-NTB) and two independent pYES2-NTB-*FoCP* transformants grew comparably at all dilution levels (10^0^ to 10^−3^). In the presence of 1 mM H_2_O_2_, the empty-vector control failed to grow at the 10^−3^ dilution, whereas both *FoCP*-expressing transformants grew at all four dilutions. At 3 mM H_2_O_2_, the empty-vector control showed no visible growth at any dilution, whereas both pYES2-NTB-*FoCP* transformants grew at all dilutions, although colony numbers were markedly reduced at the 10^−2^ and 10^−3^ dilutions (Figure 8). These results indicate that heterologous expression of *FoCP* enhances yeast growth under H_2_O_2_ stress, providing functional evidence that *FoCP* can confer oxidative-stress tolerance in a heterologous system.

Having evaluated *FoCP* as an H_2_O_2_-responsive representative, we independently used GO and KEGG enrichment, Fol ortholog-based PPI network analysis, and co-expression analysis to prioritize additional peroxidases potentially associated with infection-related processes.

### 3.10. GO and KEGG Enrichment Analysis

**Figure 9:**
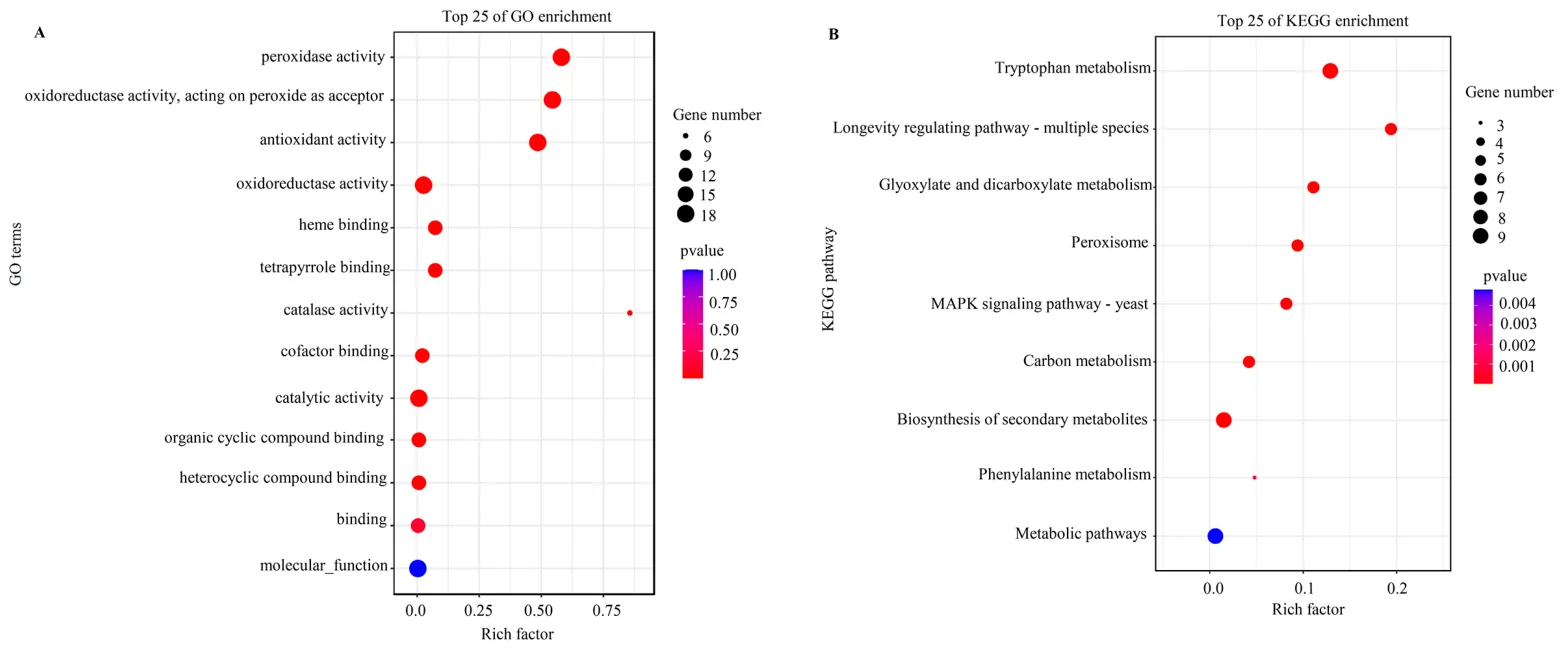
Functional enrichment analysis of *Fusarium oxysporum* f. sp. *cubense* race 4 peroxidase genes based on Gene Ontology (GO) terms and KEGG pathways. (**A**) GO enrichment analysis of 18 infection-associated peroxidase genes; (**B**) KEGG pathway enrichment analysis of nine peroxidase genes with assigned KEGG orthologs. The KEGG results therefore represent a more restricted subset of the identified peroxidase family.

GO enrichment analysis revealed that the 18 peroxidase genes represented in the infection-stage transcriptome were significantly overrepresented in molecular function terms related to peroxidase activity, oxidoreductase activity acting on peroxide as an acceptor, and antioxidant activity, with all genes annotated to these categories, while no significant enrichment was detected in the biological-process or cellular-component categories (Figure 9A, Table S9). This enrichment pattern indicates strong functional conservation in peroxide-related catalytic activities within the peroxidase gene family. In addition, significant enrichment of heme binding, tetrapyrrole binding, and cofactor binding terms was consistent with the biochemical characteristics of heme-dependent peroxidases.

KEGG pathway enrichment analysis was based on a subset of nine peroxidase genes with assigned KEGG orthologs and therefore reflected a more restricted functional scope. These genes were significantly enriched in pathways related to tryptophan metabolism, peroxisomes, glyoxylate and dicarboxylate metabolism, and the MAPK signaling pathway, as well as broader metabolic categories such as carbon metabolism and biosynthesis of secondary metabolites (Figure 9B, Table S10). Notably, KEGG enrichment mainly reflected catalase and catalase–peroxidase genes, as other peroxidase families lacked KEGG KO assignments and were excluded from pathway mapping. In contrast, GO enrichment captured the shared molecular functions of the analyzed peroxidase genes. Together, these results highlight the complementary nature of GO- and KEGG-based analyses, although the KEGG results should not be generalized to all members of the Foc4 peroxidase family.

### 3.11. Fol Ortholog-Based PPI Network and Co-Expression Analyses of Candidate Peroxidases

**Figure 10:**
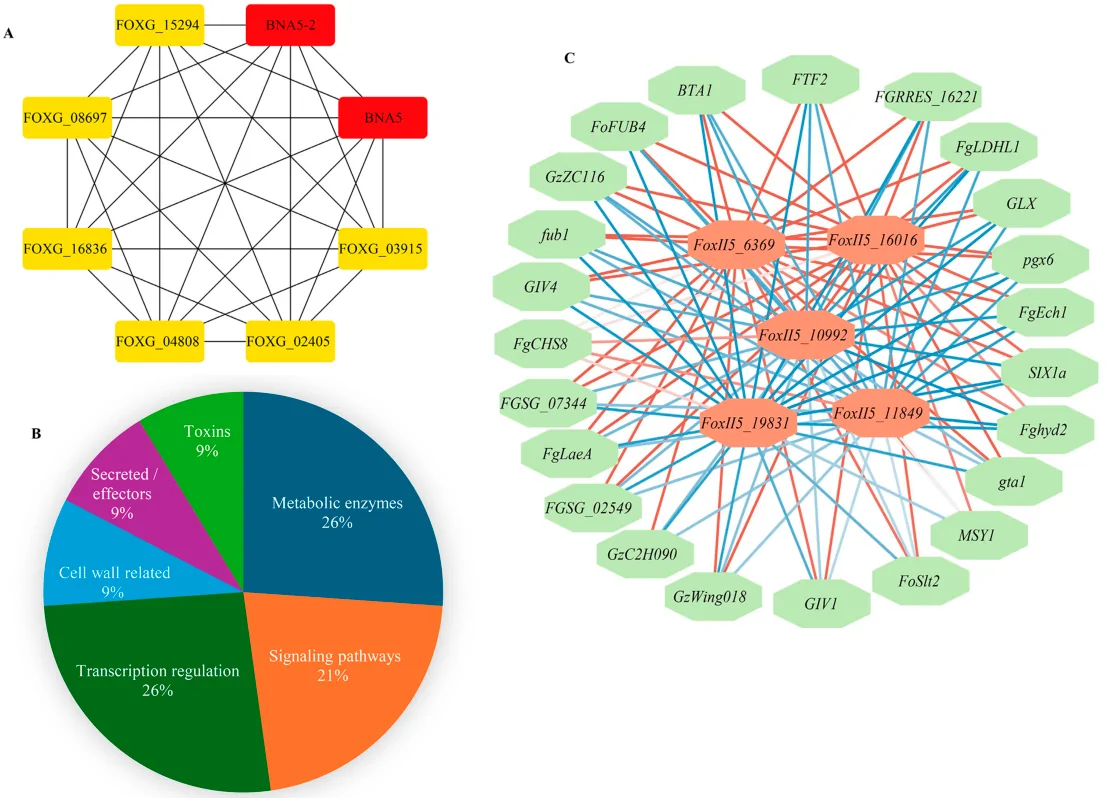
Network prioritization of *Fusarium oxysporum* f. sp. *cubense* race 4 (Foc4) peroxidases and their co-expression with pathogenicity-related genes. (**A**) STRING-predicted association network of *F. oxysporum* f. sp. *lycopersici* homologs of 18 Foc4 peroxidases. Eight central nodes were identified, of which five corresponding Foc4 genes expressed during banana infection were selected for co-expression analysis. (**B**) Functional classification of 23 Foc4 pathogenicity-related genes retrieved from the Pathogen–Host Interactions (PHI) database; (**C**) Pearson correlations between the five network-prioritized peroxidases and the 23 pathogenicity-related genes. Red and blue indicate positive and negative correlations, respectively, with color intensity representing correlation strength.

Independently of the H_2_O_2_-based selection of *FoCP*, a Fol ortholog-based predicted PPI network was used to prioritize Foc4 peroxidases according to network centrality. Eight hub nodes were identified, five of which corresponded to peroxidases expressed during banana infection: FOXG_02405 (homologous to FoxII5_19831), FOXG_08697 (homologous to FoxII5_10992), FOXG_15294 (homologous to FoxII5_11849), FOXG_03915 (homologous to FoxII5_16016), and FOXG_04808 (homologous to FoxII5_6369) (Figure 10A). These hub peroxidases occupied central positions in the predicted network, suggesting potential functional importance.

To further investigate their potential associations with pathogenicity-related expression programs, we performed co-expression analysis of these five peroxidase genes with 23 pathogenicity-related genes from the PHI database. These 23 PHI genes were functionally classified into six categories: metabolic enzymes (26.1%), signaling pathways (21.7%), transcription regulation (26.1%), cell wall-related functions (8.7%), secreted proteins/effectors (8.7%), and toxins (8.7%) (Figure 10B).

Pearson correlation analysis revealed distinct co-expression patterns among the five peroxidases (Figure 10C, Table S11). Notably, *FoxII5_16016* showed strong positive correlations with 22 of the 23 PHI genes, whereas *FoxII5_6369* showed strong positive correlations with all 23 PHI genes (r ≥ 0.7). In contrast, *FoxII5_19831*, *FoxII5_10992*, and *FoxII5_11849* exhibited strong negative correlations with the majority of the PHI genes (r ≤ −0.7). These contrasting patterns suggest that different members of the peroxidase family may be associated with distinct pathogenicity-related expression programs during infection. Given that these correlations were calculated from only three time points (18, 32, and 56 hpi), they should be regarded as exploratory descriptive associations; no *p* values or multiple-testing corrections were calculated.

## 4. Discussion

This study provides the first genome-wide identification of the peroxidase gene family in Foc4 and characterizes its domain architecture, regulatory context, expression dynamics, functional properties, and evolutionary constraints. Comparative analyses with other fungal pathogens further clarify the potential contributions of Foc4 peroxidases to ROS detoxification and infection-associated processes.

Conserved-motif analysis identified 10 motifs showing clade-specific distributions across four phylogenetic groups. Motif 3 was specific to Group I, motifs 4 and 9 were enriched in Group II, and motifs 5, 8, and 10 were concentrated in Group IV. This clade-specific motif distribution indicates divergence in key structural regions among peroxidase clades, consistent with functional specialization. Similar patterns of phylogenetic and functional divergence have been reported for heme peroxidases [28,29]. Accordingly, variation in these structural units may contribute to differences among subfamilies in catalytic properties, substrate preferences, and/or subcellular localization. Group III members displayed greater motif complexity, suggesting roles that may extend beyond canonical ROS scavenging and may involve additional biological processes. Notably, FoxII5_14434 contains a DyP-type peroxidase domain with similarity to EfeB/YfeX-like proteins, although typical DyP conserved motifs were not readily detected. This protein may therefore represent a divergent or atypical DyP peroxidase, or may reflect an incomplete or misannotated gene model. Further structural and functional analyses are needed to clarify this possibility. Eighteen infection-associated peroxidases were significantly enriched for “peroxidase activity,” “oxidoreductase activity,” and “antioxidant activity” (Figure 9A), consistent with their predicted roles in H_2_O_2_ decomposition and redox homeostasis. Enrichment of heme-binding and cofactor-binding terms further supports their classification as heme-dependent enzymes.

Promoter analysis identified 695 cis-acting elements, primarily associated with light responsiveness, methyl jasmonate (MeJA), abscisic acid (ABA), and anaerobic induction. This distribution suggests that Foc4 peroxidase expression may respond to diverse environmental and host-related signals. MeJA can inhibit fungal growth by perturbing the cAMP–PKA pathway [30], whereas ABA can also be produced by fungi and contribute to host adaptation [31]. Thus, host-associated hormonal cues may influence the transcriptional regulation of Foc4 peroxidases during infection, although this possibility requires experimental verification.

Time-course expression profiling during infection grouped peroxidase genes into early-induced, late-induced, and low-expression clusters, suggesting a temporally coordinated antioxidant program. Early-induced genes may help counter the initial host oxidative burst, paralleling the role of *KatG2* in *F. graminearum* [8], whereas late-induced genes may respond to oxidative stress associated with later stages of vascular colonization [32]. Integration of infection-stage and H_2_O_2_-induced expression profiles further revealed heterogeneous responses: some genes were induced under both conditions, whereas others responded specifically to H_2_O_2_ treatment. These patterns suggest that Foc4 peroxidases may participate in both ROS detoxification and stage-specific infection-related responses.

Heterologous expression of *FoCP* (*FoxII5_1335143*) in *S. cerevisiae* enhanced growth under H_2_O_2_ stress, with the transformants retaining growth at 3 mM H_2_O_2_, whereas the empty-vector control showed no visible growth. This finding is consistent with previous studies showing that deletion of *FvCP01* and *FvCP02* increased H_2_O_2_ sensitivity in *F. verticillioides* [33], whereas deletion of *FCA7* impaired oxidative-stress tolerance in *F. graminearum* [22]. Together, these results suggest that *FoCP* can enhance oxidative-stress tolerance in yeast, although its direct enzymatic activity was not determined. *FoCP* expression in the transformed yeast was not confirmed at the transcript or protein level, which represents a limitation of the heterologous-expression assay.

*FoCP* was not among the five peroxidases prioritized by the Fol ortholog-based network analysis because the two selection procedures addressed different questions. *FoCP* was selected based on its rapid H_2_O_2_-induced expression and catalase–peroxidase annotation to assess the function of a representative Foc4 enzyme in a heterologous system. By contrast, FoxII5_19831, FoxII5_10992, FoxII5_11849, FoxII5_16016, and FoxII5_6369 were selected based on predicted network centrality and infection-stage expression. Thus, these candidates arose from two independent selection strategies, and the functions of the five network-prioritized genes require further validation in Foc4.

The Foc4 peroxidase family showed limited gene expansion and retained a conserved functional repertoire. Phylogenetic analyses indicated clear orthologous relationships between Foc4 peroxidases and those of Fol, *M. oryzae*, and *F. graminearum*, with broadly stable family sizes across species. Ka/Ks analysis further showed strong purifying selection across all members (Ka/Ks = 0–0.30), indicating conservation of core functional properties.

Synteny analysis showed that genomic regions harboring these genes are highly conserved between pathogenic Foc4 and Fol, whereas the non-pathogenic strain Fo47 exhibits pronounced rearrangements. This pattern may be interpreted in the context of the “two-speed” genome model, in which a conserved core genome underpins essential biological processes while a rapidly evolving accessory genome accommodates mobile elements, including host-adaptation effectors [34,35,36]. The conserved synteny of the peroxidase genes therefore suggests that they are mainly located in conserved genomic regions and may represent core functional components. However, conserved genomic location does not necessarily mean that these genes function solely as housekeeping factors. Instead, the infection- and H_2_O_2_-responsive expression patterns suggest that regulatory variation may contribute to their functional diversification. In the context of ROS detoxification, differences in promoter elements and expression timing may be more important than changes in gene number, allowing stage-specific responses to host oxidative bursts during infection [8,19].

KEGG enrichment analysis further indicated potential involvement in the “peroxisome,” “tryptophan metabolism,” and “MAPK signaling pathway” categories (Figure 9B). These pathways are potentially related to ROS homeostasis, cellular metabolism, and signal transduction, respectively. Co-expression analysis provided further support for potential links between selected peroxidases and infection-associated processes. *FoxII5_16016* showed strong positive correlations with 22 of the 23 pathogenicity-related genes, whereas *FoxII5_6369* showed strong positive correlations with all 23 genes, including the fusaric acid biosynthesis genes *fub1* and *FoFUB4* [37,38], the MAPK FoSlt2 [39], the transcription factor FTF2 [40], the global regulator LaeA [41], and the effector Six1a [42]. These associations suggest that the two peroxidase genes may occupy important positions in co-expression networks related to toxin biosynthesis, signaling, and infection-associated processes, although they do not establish direct regulatory relationships. Conversely, *FoxII5_11849* showed negative correlations with most pathogenicity-related genes, possibly reflecting stage-specific inverse expression patterns. Similar associations between oxidative-stress responses and pathogenicity have been reported for MoAP1 in *M. oryzae* [43], while deletion of *FCA7* in *F. graminearum* affects both oxidative-stress sensitivity and mycotoxin production [22].

**Figure 11:**
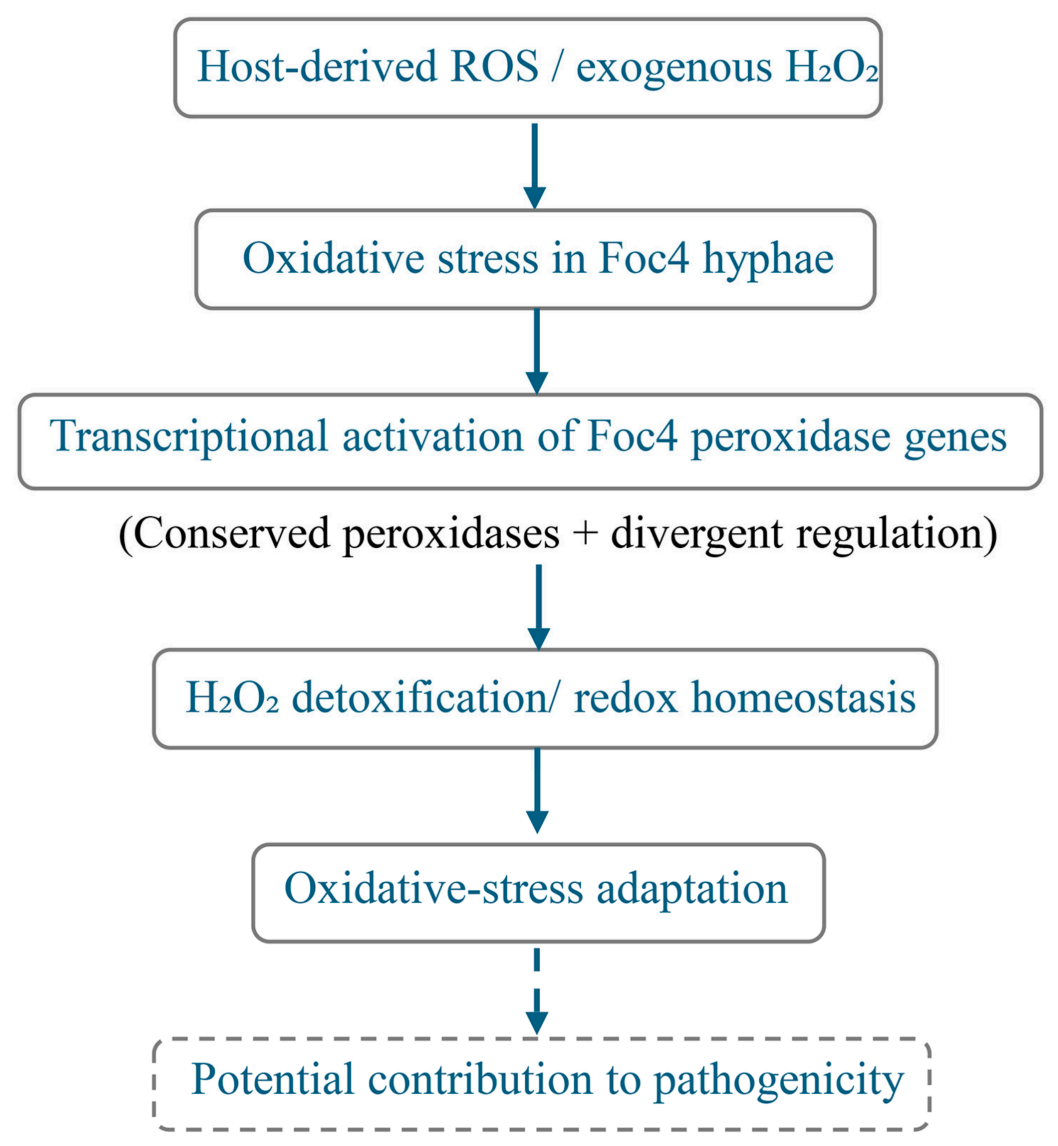
Proposed working model of *Fusarium oxysporum* f. sp. *cubense* race 4 (Foc4) peroxidases in oxidative-stress adaptation. Host-derived ROS during infection and exogenous H_2_O_2_ treatment induce condition-specific transcriptional responses in evolutionarily conserved Foc4 peroxidases. A representative catalase–peroxidase, *FoCP*, enhances H_2_O_2_ tolerance when heterologously expressed in yeast, whereas independently identified network-prioritized peroxidases are associated with pathogenicity-related expression programs. These findings suggest that regulatory diversification within the conserved family may contribute to redox homeostasis and oxidative-stress adaptation. Solid arrows denote relationships supported by expression, enrichment, or yeast tolerance data, whereas dashed arrows indicate putative links to pathogenicity-related processes that require experimental validation in Foc4.

Based on these findings, we propose a working model in which diversified transcriptional regulation of conserved peroxidases contributes to oxidative-stress adaptation in Foc4 (Figure 11). Host-derived ROS may impose oxidative pressure on invading hyphae, whereas exogenous H_2_O_2_ treatment partially mimics this stress in vitro. Specific peroxidases may contribute to ROS homeostasis through condition- and stage-dependent expression. The *FoCP* experiment demonstrates that a representative Foc4 catalase–peroxidase confers H_2_O_2_ tolerance in a heterologous system, whereas the potential virulence roles of the network-prioritized peroxidases remain hypothetical and require genetic validation in Foc4. Overall, the relatively stable peroxidase gene number among the compared *Fusarium* species suggests that transcriptional regulation may contribute more to the functional diversification of Foc4 peroxidases than gene-family expansion.

In summary, this study delineates the composition, evolutionary conservation, regulatory characteristics, and expression dynamics of the Foc4 peroxidase gene family. The results support potential roles for selected peroxidases in oxidative-stress adaptation and infection-associated expression programs, while their direct contributions to pathogenicity remain to be tested by genetic, biochemical, and plant infection assays.

## 5. Conclusions

This study provides the first genome-wide characterization of the Foc4 peroxidase gene family and shows that it is evolutionarily conserved, under strong purifying selection, and has experienced limited lineage-specific expansion. Integrated expression, functional enrichment, and network analyses revealed regulatory diversification among Foc4 peroxidases and identified several hub genes potentially associated with oxidative-stress responses and pathogenicity-related expression programs. These findings suggest that Foc4 may cope with host-derived oxidative stress primarily through regulatory diversification of a conserved antioxidant repertoire rather than gene-family expansion, and heterologous expression of *FoCP* suggested that it can enhance oxidative-stress tolerance in yeast. The hub peroxidases identified by network analysis represent promising candidates for functional validation and disease-control studies.

## Figures and Tables

**Table 1:** Basic information on peroxidase gene family members of *Fusarium oxysporum* f. sp. *cubense* race 4.

Gene ID	Protein Length (aa)	Molecular Weight (Da)	Theoretical pI	Subcellular Localization	Conserved Domain
*FoxII5_11579*	435	46,983.50	5.94	Extracellular	Peroxidase_2
*FoxII5_1372619*	731	79,871.12	6.46	Extracellular	Peroxidase_2
*FoxII5_14536*	452	49,316.83	5.94	Extracellular	Peroxidase_2 superfamily
*FoxII5_1349250*	457	49,582.46	8.97	Peroxisomal	Peroxidase_2 superfamily
*FoxII5_16098*	231	24,882.97	4.99	Extracellular	Peroxidase_2
*FoxII5_1252136*	698	77,938.09	5.98	Extracellular	Peroxidase_2
*FoxII5_7493*	264	29,704.73	7.11	Mitochondrial	Peroxidase_2
*FoxII5_10492*	270	29,919.11	7.02	Nuclear	Peroxidase_2 superfamily
*FoxII5_19678*	271	30,522.01	6.00	Cytoskeleton	Peroxidase_2
*FoxII5_6369*	374	40,990.69	6.16	Cytoplasmic	catalase-like (srpA_like)
*FoxII5_10992*	578	65,427.03	6.21	Nuclear	catalase_fungal
*FoxII5_1210918*	578	65,703.67	6.01	Nuclear	Catalase
*FoxII5_18281*	506	57,195.35	6.42	Cytoplasmic	catalase_fungal
*FoxII5_16016*	487	54,879.87	6.12	Cytoplasmic	catalase_fungal
*FoxII5_11849*	492	54,945.19	6.36	Cytoplasmic	catalase_like superfamily
*FoxII5_19831*	738	82,702.02	5.90	Cytoplasmic	katE superfamily
*FoxII5_14434*	361	39,933.06	5.53	Mitochondrial	DyP-type peroxidase (EfeB/YfeX-like)
*FoxII5_1200130*	358	40,075.87	8.24	Mitochondrial	ascorbate_peroxidase
*FoxII5_9926*	331	36,552.73	5.13	Peroxisomal	ascorbate_peroxidase
*FoxII5_1369499*	512	55,366.14	8.06	Extracellular	plant_peroxidase_like superfamily
*FoxII5_17640*	530	57,338.88	8.88	Extracellular	plant_peroxidase_like superfamily
*FoxII5_20543*	781	85,923.92	6.39	Extracellular	KatG
*FoxII5_14360*	777	85,058.02	6.57	Extracellular	KatG
*FoxII5_1335143*	801	88,540.06	5.65	Mitochondrial	KatG

Note: Gene IDs shown in this table are standardized identifiers used throughout this study and correspond to the original gene identifiers in the genome annotation (GFF3) file (e.g., *FoxII5_11579* corresponds to *gene-NOF04DRAFT_11579*).

## Data Availability

The raw RNA-seq data generated from Foc4 under hydrogen peroxide stress in this study have been deposited in the Genome Sequence Archive at the National Genomics Data Center (https://ngdc.cncb.ac.cn/gsa/, accessed on 7 June 2026) under accession number CRA044072.

## Supplementary Materials

The following supporting information can be downloaded at: https://www.mdpi.com/article/10.3390/jof12090653/s1, Figure S1: Sequence logos of the ten conserved motifs identified in *Fusarium oxysporum* f. sp. *cubense* race 4 peroxidase proteins; Table S1: Non-heme, thiol-dependent peroxide-reducing enzymes and related proteins identified in *Fusarium oxysporum* f. sp. *cubense* race 4; Table S2: Coding, protein, and 2-kb upstream promoter sequences of the 24 *Fusarium oxysporum* f. sp. *cubense* race 4 peroxidase genes; Table S3: Pairwise Ka/Ks values of orthologous peroxidase genes between *Fusarium oxysporum* f. sp. *cubense* race 4 (Foc4) and *F. oxysporum* f. sp. *lycopersici* (Fol), including the additional comparison between FoxII5_6369 and its *F. graminearum* ortholog FGSG_02217; Table S4: Details of the cis-acting regulatory elements identified in the 2-kb upstream regions of *Fusarium oxysporum* f. sp. *cubense* race 4 peroxidase genes; Table S5: FPKM values of peroxidase genes at 18, 32, and 56 h post-infection during banana infection; Table S6: FPKM values of peroxidase genes at six H_2_O_2_ treatment time points; Table S7: Primer sequences used for qRT-PCR and vector construction in this study; Table S8: Expression stability ranking of candidate reference genes under H_2_O_2_ stress; Table S9: Gene Ontology enrichment analysis of 18 infection-associated peroxidase genes; Table S10: KEGG enrichment analysis of nine peroxidase genes with assigned KEGG orthologs; Table S11: Pearson correlation coefficient matrix showing expression correlations between five network-prioritized peroxidase genes and 23 pathogenicity-related genes from the PHI database.

## References

[1] Bubici G., Kaushal M., Prigigallo M.I., Gomez-Lama Cabanas C., Mercado-Blanco J. (2019). Biological control agents against Fusarium wilt of banana. Front. Microbiol..

[2] Liu L., Du Z., Xiao X., Cheng S., Zhong Y., Li Z., Zeng S., Lin H., Fu Q., Ding Z. (2026). Histidinol-phosphate phosphatase FoHis2 is essential for growth, stress responses, and full virulence in *Fusarium oxysporum* f. sp. *cubense*. J. Fungi.

[3] Ploetz R.C. (2015). Fusarium wilt of banana. Phytopathology.

[4] Su H.-J., Hwang S.-C., Ko W.-H. (1986). Fusarial wilt of Cavendish bananas in Taiwan. Plant Dis..

[5] Anthony K.K., George D.S., Baldev Singh H.K., Fung S.M., Santhirasegaram V., Razali Z., Somasundram C. (2017). Reactive oxygen species activity and antioxidant properties of *Fusarium* infected bananas. J. Phytopathol..

[6] Park J., Son H. (2024). Antioxidant systems of plant pathogenic fungi: Functions in oxidative stress response and their regulatory mechanisms. Plant Pathol. J..

[7] Mittler R., Zandalinas S.I., Fichman Y., Van Breusegem F. (2022). Reactive oxygen species signalling in plant stress responses. Nat. Rev. Mol. Cell Biol..

[8] Guo Y., Yao S., Yuan T., Wang Y., Zhang D., Tang W. (2019). The spatiotemporal control of KatG2 catalase-peroxidase contributes to the invasiveness of *Fusarium graminearum* in host plants. Mol. Plant Pathol..

[9] Mandal S., Mitra A., Mallick N. (2008). Biochemical characterization of oxidative burst during interaction between *Solanum lycopersicum* and *Fusarium oxysporum* f. sp. *lycopersici*. Physiol. Mol. Plant Pathol..

[10] Segal L.M., Wilson R.A. (2018). Reactive oxygen species metabolism and plant-fungal interactions. Fungal Genet. Biol..

[11] Marschall R., Tudzynski P. (2016). Reactive oxygen species in development and infection processes. Semin. Cell Dev. Biol..

[12] Mir A.A., Park S.Y., Abu Sadat M., Kim S., Choi J., Jeon J., Lee Y.H. (2015). Systematic characterization of the peroxidase gene family provides new insights into fungal pathogenicity in *Magnaporthe oryzae*. Sci. Rep..

[13] de Oliveira M.A., Tairum C.A., Netto L.E.S., de Oliveira A.L.P., Aleixo-Silva R.L., Cabrera V.I.M., Breyer C.A., Dos Santos M.C. (2021). Relevance of peroxiredoxins in pathogenic microorganisms. Appl. Microbiol. Biotechnol..

[14] Zhang S., Wang J., Li B., Zang J., Cao H., Xing J., Dong J., Zhang K. (2025). Molecular characterisation of the peroxidase gene family in Botrytis cinerea and the role of BcPRD7 in virulence. Mol. Plant Pathol..

[15] Sugano Y., Yoshida T. (2021). DyP-type peroxidases: Recent advances and perspectives. Int. J. Mol. Sci..

[16] Qin X., Luo H., Zhang X., Yao B., Ma F., Su X. (2018). Dye-decolorizing peroxidases in Irpex lacteus combining the catalytic properties of heme peroxidases and laccase play important roles in ligninolytic system. Biotechnol. Biofuels.

[17] Poljovka A., Musil M., Bednář D., Chovanová K., Bauerová-Hlinková V., Bellová J., Kohútová L., Baráth P., Zámocký M. (2023). Comparison of fungal thermophilic and mesophilic catalase–peroxidases for their antioxidative properties. Antioxidants.

[18] Li G., Fan A., Peng G., Keyhani N.O., Xin J., Cao Y., Xia Y. (2017). A bifunctional catalase-peroxidase, MakatG1, contributes to virulence of *Metarhizium acridum* by overcoming oxidative stress on the host insect cuticle. Environ. Microbiol..

[19] Liu M., Guo Z., Hu J., Chen Y., Chen F., Chen W., Wang W., Ye B., Yang Z., Li G. (2025). The multifunctional ascorbate peroxidase MoApx1 secreted by *Magnaporthe oryzae* mediates the suppression of rice immunity. Plant Cell.

[20] Liu F., Guo L., Luo Y., Li J., Zhou Y., Wang J., Huang X., Tan X., Fu M., Yu B. (2025). Peroxiredoxin PrxA and thioredoxin TrxA mediate the redox signal to the transcription factor NapA in the fungus *Aspergillus nidulans*. Int. J. Biol. Macromol..

[21] Mendoza-Martinez A.E., Sanchez O., Aguirre J. (2023). The role of peroxiredoxins PrxA and PrxB in the antioxidant response, carbon utilization and development in *Aspergillus nidulans*. Fungal Biol..

[22] Lee Y., Son H., Shin J.Y., Choi G.J., Lee Y.W. (2018). Genome-wide functional characterization of putative peroxidases in the head blight fungus *Fusarium graminearum*. Mol. Plant Pathol..

[23] Zhang Y., Liu S., Mostert D., Yu H., Zhuo M., Li G., Zuo C., Haridas S., Webster K., Li M. (2024). Virulence of banana wilt-causing fungal pathogen *Fusarium oxysporum* tropical race 4 is mediated by nitric oxide biosynthesis and accessory genes. Nat. Microbiol..

[24] Chen C., Wu Y., Li J., Wang X., Zeng Z., Xu J., Liu Y., Feng J., Chen H., He Y. (2023). TBtools-II: A “one for all, all for one” bioinformatics platform for biological big-data mining. Mol. Plant.

[25] Savelli B., Li Q., Webber M., Jemmat A.M., Robitaille A., Zamocky M., Mathe C., Dunand C. (2019). RedoxiBase: A database for ROS homeostasis regulated proteins. Redox Biol..

[26] Wang Y., Tang H., Wang X., Sun Y., Joseph P.V., Paterson A.H. (2024). Detection of colinear blocks and synteny and evolutionary analyses based on utilization of MCScanX. Nat. Protoc..

[27] Lescot M., Dehais P., Thijs G., Marchal K., Moreau Y., Van de Peer Y., Rouze P., Rombauts S. (2002). PlantCARE, a database of plant cis-acting regulatory elements and a portal to tools for in silico analysis of promoter sequences. Nucleic Acids Res..

[28] Faiza M., Huang S., Lan D., Wang Y. (2019). New insights on unspecific peroxygenases: Superfamily reclassification and evolution. BMC Evol. Biol..

[29] Zamocky M. (2004). Phylogenetic relationships in class I of the superfamily of bacterial, fungal, and plant peroxidases. Eur. J. Biochem..

[30] Duan K., Qin S., Cui F., Zhao L., Huang Y., Xu J.R., Wang G. (2025). MeJA inhibits fungal growth and DON toxin production by interfering with the cAMP-PKA signaling pathway in the wheat scab fungus *Fusarium graminearum*. mBio.

[31] Wang Y., Zhou J., Zhong J., Luo D., Li Z., Yang J., Shu D., Tan H. (2018). Cys_2_His_2_ zinc finger transcription factor BcabaR1 positively regulates abscisic acid production in *Botrytis cinerea*. Appl. Environ. Microbiol..

[32] de Quadros F.M., Garcés-Fiallos F.R., de Freitas M.B., Bouzon Z.L., Barcelos-Oliveira J.L., Stadnik M.J. (2021). Dual role of H_2_O_2_ in late stages of root colonization of resistant and susceptible bean plants by *Fusarium oxysporum* f. sp. *phaseoli*. Physiol. Mol. Plant Pathol..

[33] Gao S., Gold S.E., Glenn A.E. (2018). Characterization of two catalase-peroxidase-encoding genes in *Fusarium verticillioides* reveals differential responses to in vitro versus in planta oxidative challenges. Mol. Plant Pathol..

[34] Madhushan A., Weerasingha D.B., Ilyukhin E., Taylor P.W.J., Ratnayake A.S., Liu J.K., Maharachchikumbura S.S.N. (2025). From natural hosts to agricultural threats: The evolutionary journey of phytopathogenic fungi. J. Fungi.

[35] van Westerhoven A.C., Aguilera-Galvez C., Nakasato-Tagami G., Shi-Kunne X., Martinez de la Parte E., Chavarro-Carrero E., Meijer H.J.G., Feurtey A., Maryani N., Ordonez N. (2024). Segmental duplications drive the evolution of accessory regions in a major crop pathogen. New Phytol..

[36] Moller M., Stukenbrock E.H. (2017). Evolution and genome architecture in fungal plant pathogens. Nat. Rev. Microbiol..

[37] Phasha M.M., Wingfield B.D., Wingfield M.J., Coetzee M.P.A., Hammerbacher A., Steenkamp E.T. (2021). Deciphering the effect of FUB1 disruption on fusaric acid production and pathogenicity in *Fusarium circinatum*. Fungal Biol..

[38] Ding Z., Yang L., Wang G., Guo L., Liu L., Wang J., Huang J. (2018). Fusaric acid is a virulence factor of *Fusarium oxysporum* f. sp. *cubense* on banana plantlets. Trop. Plant Pathol..

[39] Ding Z., Li M., Sun F., Xi P., Sun L., Zhang L., Jiang Z. (2015). Mitogen-activated protein kinases are associated with the regulation of physiological traits and virulence in *Fusarium oxysporum* f. sp. *cubense*. PLoS ONE.

[40] Nino-Sanchez J., Casado-Del Castillo V., Tello V., De Vega-Bartol J.J., Ramos B., Sukno S.A., Diaz Minguez J.M. (2016). The FTF gene family regulates virulence and expression of SIX effectors in *Fusarium oxysporum*. Mol. Plant Pathol..

[41] Moon H., Lee M.K., Bok I., Bok J.W., Keller N.P., Yu J.H. (2023). Unraveling the gene regulatory networks of the global regulators VeA and LaeA in *Aspergillus nidulans*. Microbiol. Spectr..

[42] Widinugraheni S., Nino-Sanchez J., van der Does H.C., van Dam P., Garcia-Bastidas F.A., Subandiyah S., Meijer H.J.G., Kistler H.C., Kema G.H.J., Rep M. (2018). A SIX1 homolog in *Fusarium oxysporum* f.sp. *cubense* tropical race 4 contributes to virulence towards Cavendish banana. PLoS ONE.

[43] Guo M., Chen Y., Du Y., Dong Y., Guo W., Zhai S., Zhang H., Dong S., Zhang Z., Wang Y. (2011). The bZIP transcription factor MoAP1 mediates the oxidative stress response and is critical for pathogenicity of the rice blast fungus *Magnaporthe oryzae*. PLoS Pathog..

